# Supplementary material for: A repositioning screen using an FGFR2 splicing reporter reveals compounds that regulate epithelial-mesenchymal transitions and inhibit growth of prostate cancer xenografts
Source: Mol Ther Methods Clin Dev. 2022 Mar 17;25:147–57. doi: 10.1016/j.omtm.2022.03.005 (PMC8971352; doi:10.1016/j.omtm.2022.03.005)
Supplement: Document S1. Figures S1–S13 [file mmc1.pdf]

OMTM, Volume *25*

Supplemental information

A repositioning screen using an FGFR2 splicing reporter reveals compounds that regulate epithelial-mesenchymal transitions and inhibit growth of prostate cancer xenografts

Ling Li, Jinxia Zheng, Megan Stevens, and Sebastian Oltean

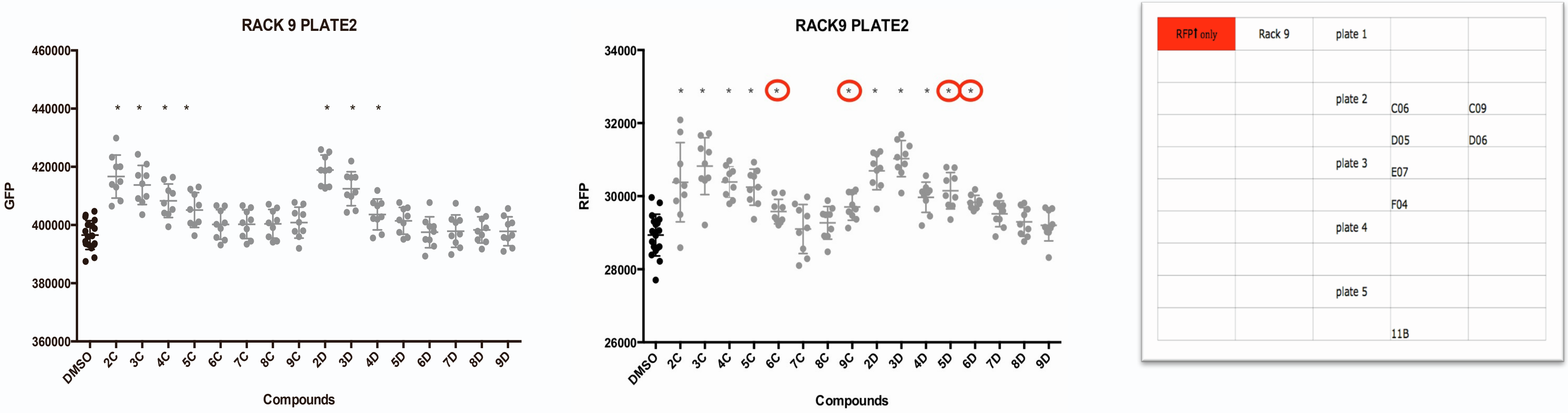

Supplementary Figure 1. Examples of readings in the primary screen, that resulted in narrowing down to 278 compounds.

Left panel represent GFP read-outs from the plate reader and data analysed by Prism.

Middle panel represent dsRED read-outs from the plate reader and data analysed by Prism, red circles marked the hits that increased dsRED without increasing GFP signal.

Right panel represent the final result, in which red means RFP increase only. X-axis represent the location in the 96-well plate. Each well represent one repeat of one chemical treatment. Each treatment had three repeats. *$p<0.05$ by One-way ANOVA using GraphPad Prism.

Primary screen result		Elimination screen	Final result
Rack 10	D07	RFP↑ only	False-positives
Rack 10	F04	RFP⬆ + GFP⬇	False-positives
Rack 13	B04	RFP↑ only	GFP↓ only
Rack 14	B05	RFP↑ only	False-positives
Rack 15	E10	GFP↓ only	RFP↑ only

Supplementary Figure 2. Examples of eliminating false-positive
Red means RFP increase only, green means GFP decrease only, and yellow means both of them in the primary screen. Rack10, compound D07 showed RFP increase in primary screen but also showed the same result in elimination-screen, so it was eliminated. Rack13 B04 showed both changes in the primary screen while only increased RFP in the elimination screen; therefore, the effect suggested by GFP decrease may be a real one, related to splicing and not a false-positive.

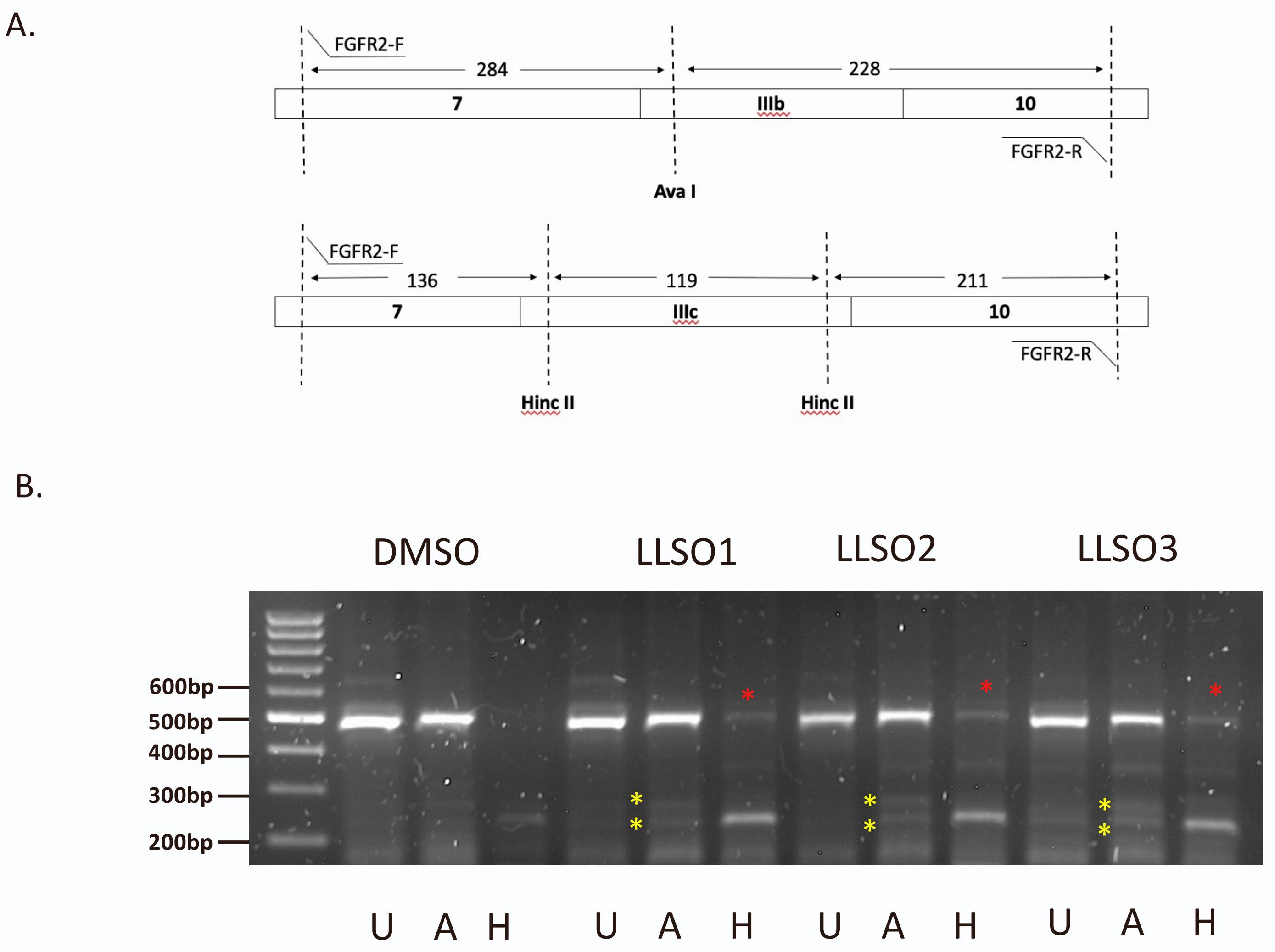

Supplementary Figure 3. The same analysis as Figure 2 (see legend) but done in PC3 prostate cancer cells instead of HEK293

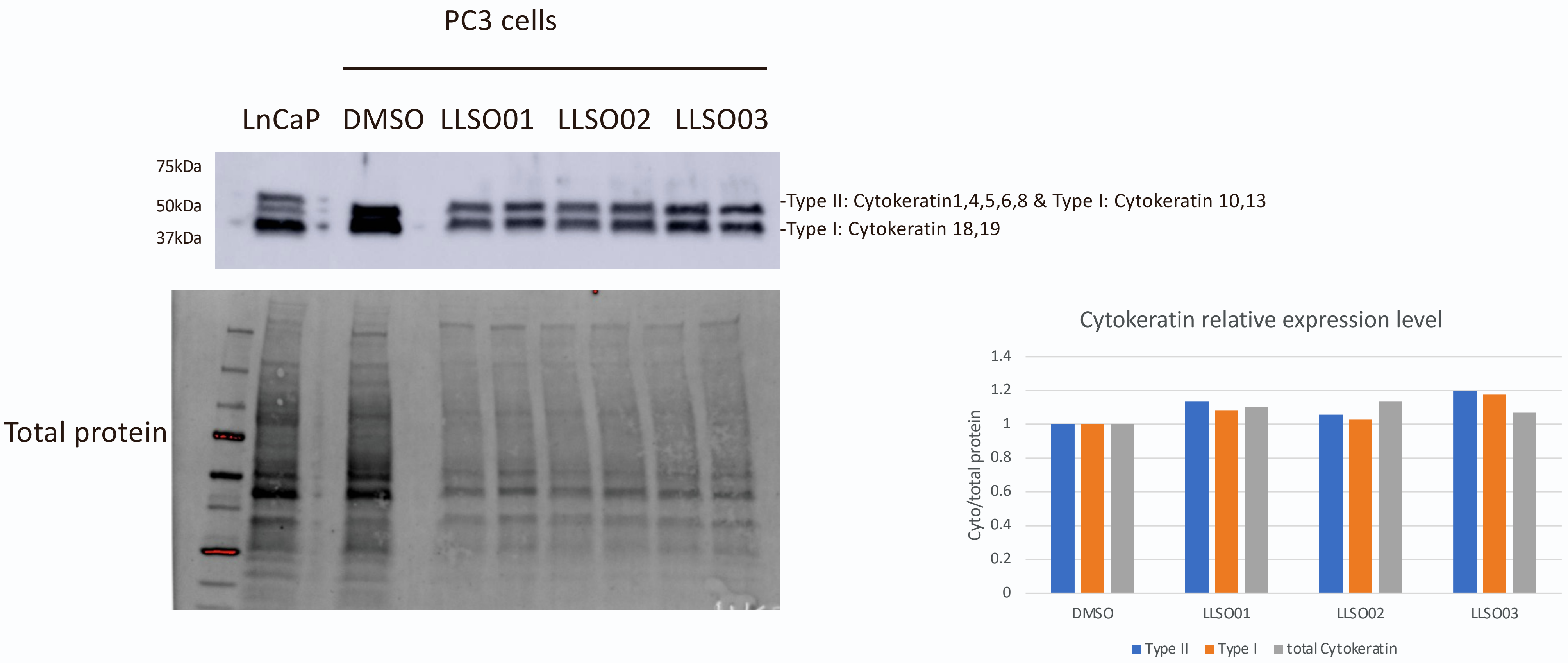

Supplementary Figure 4. Response of additional markers of EMT to LLSOs treatment - cytokeratins. Western blot analysis showing a slight increase in cytokeratins expression in PC3 cells exposed to LLSOs treatments for 48 h at 10uM (n=2 replicates, 1experiment).

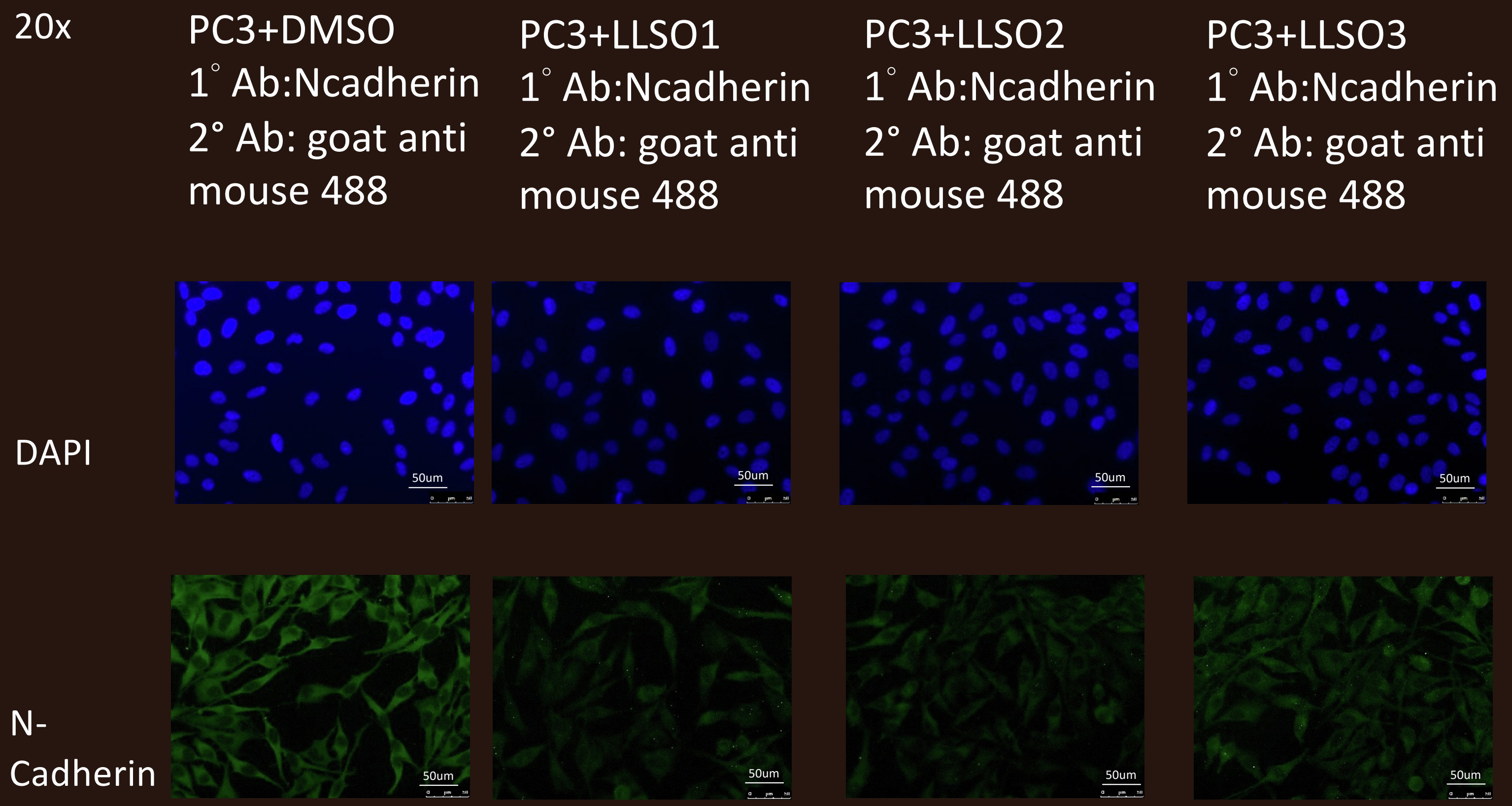

Supplementary Figure 5. Immunofluorescence analysis of PC3 cells treated with LLSO1, LLSO2 or LLSO3 at 10 uM for 48 hours shows a decrease in expression of N-cadherin. Representative examples from at least 10 microscopic fields per experiment, and experiments repeated 3 times. Pictures taken at 200 X magnification.

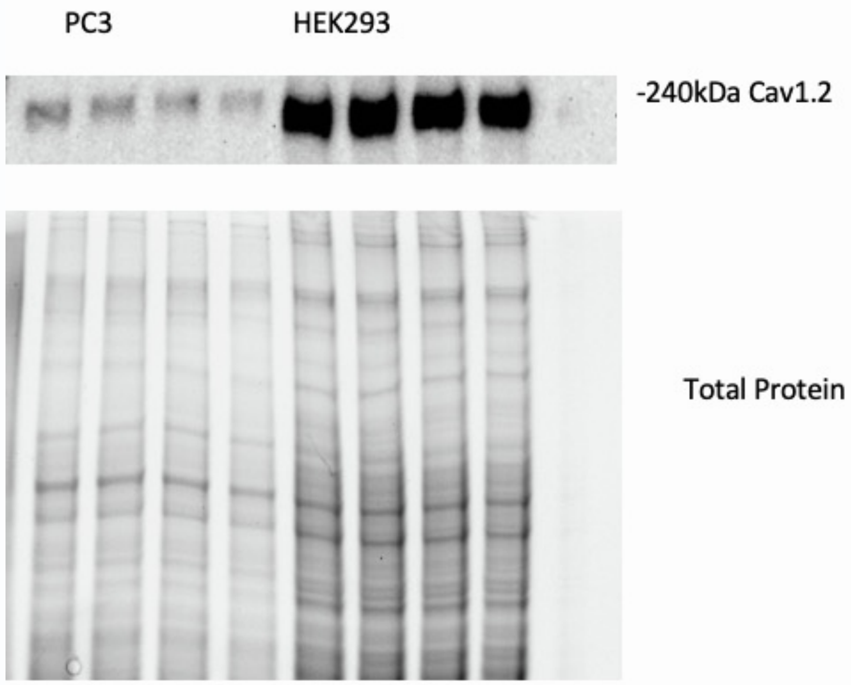

Supplementary Figure 6. L-type Ca channels are expressed in PC3 prostate cancer cells

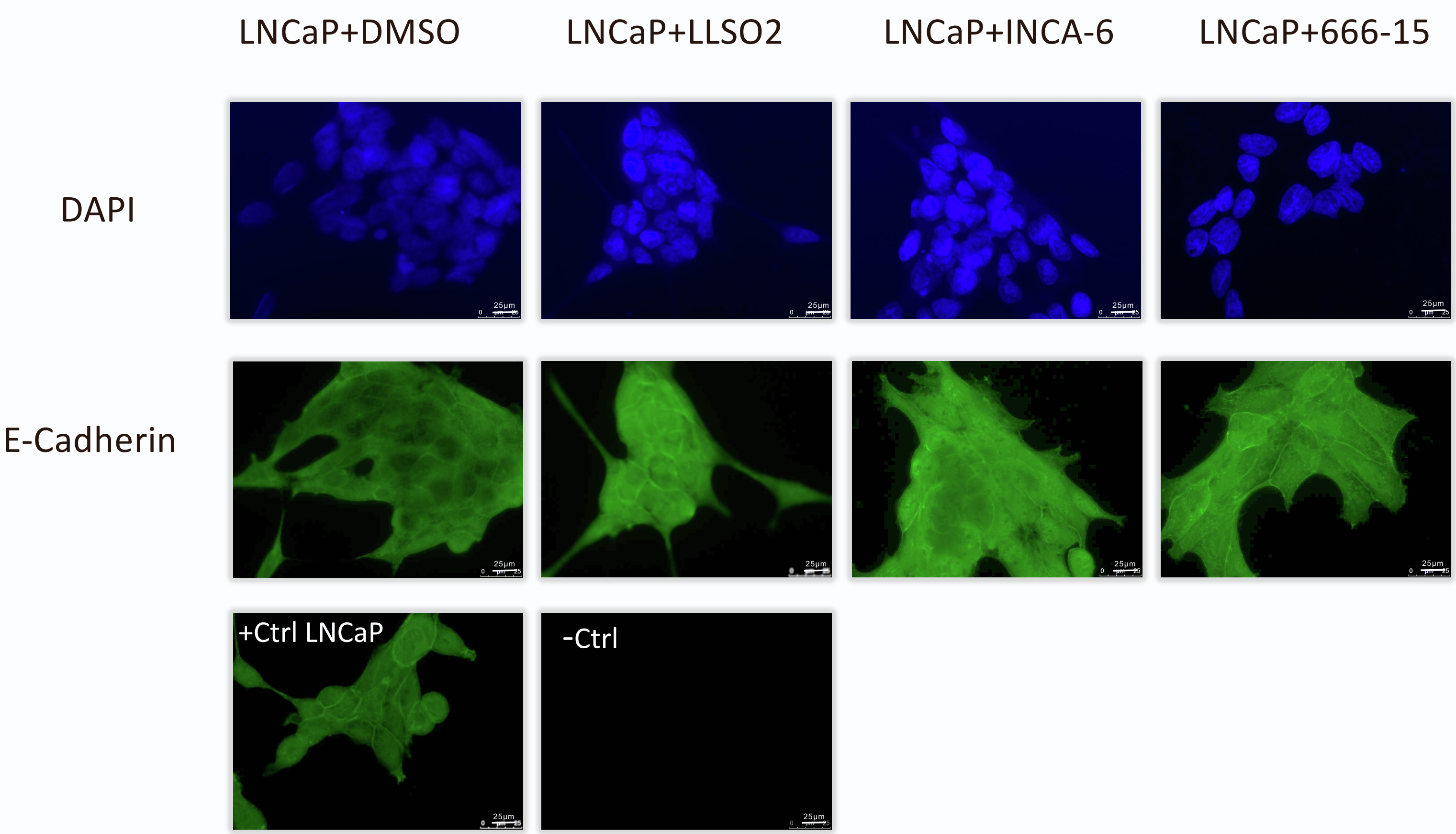

Supplementary Figure 7. LLSO02 uses NFAT and/or CREB to signal to increase E-cadherin expression in LNCaP cells
Images of LNCaP cells treated separately with 10μM of LLSO2, 10μM of INCA-6, and 10μM of 666-15
All images are taken at 20x magnification.

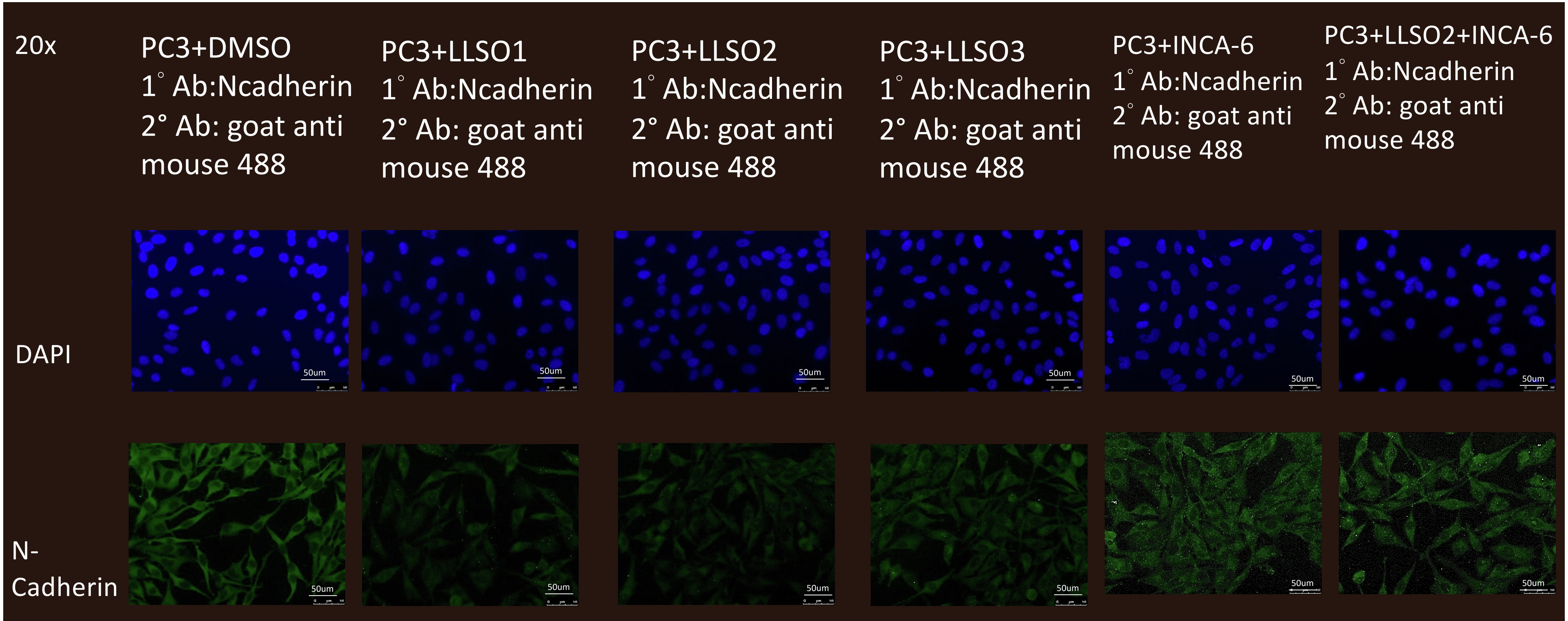

Supplementary Figure 8. Immunofluorescence analysis of PC3 cells treated with LLSO1, LLSO2 or LLSO3 at 10 uM for 48 hours shows a decrease in expression of N-cadherin (the examples shown here are the same as the ones shown in Supplementary Figure 5 above). When INCA-6 is used or INCA-6 and LLSO2, N-cadherin expression is rescued. Representative examples from at least 10 microscopic fields per experiment, and experiments repeated 3 times. Pictures taken at 200 X magnification.

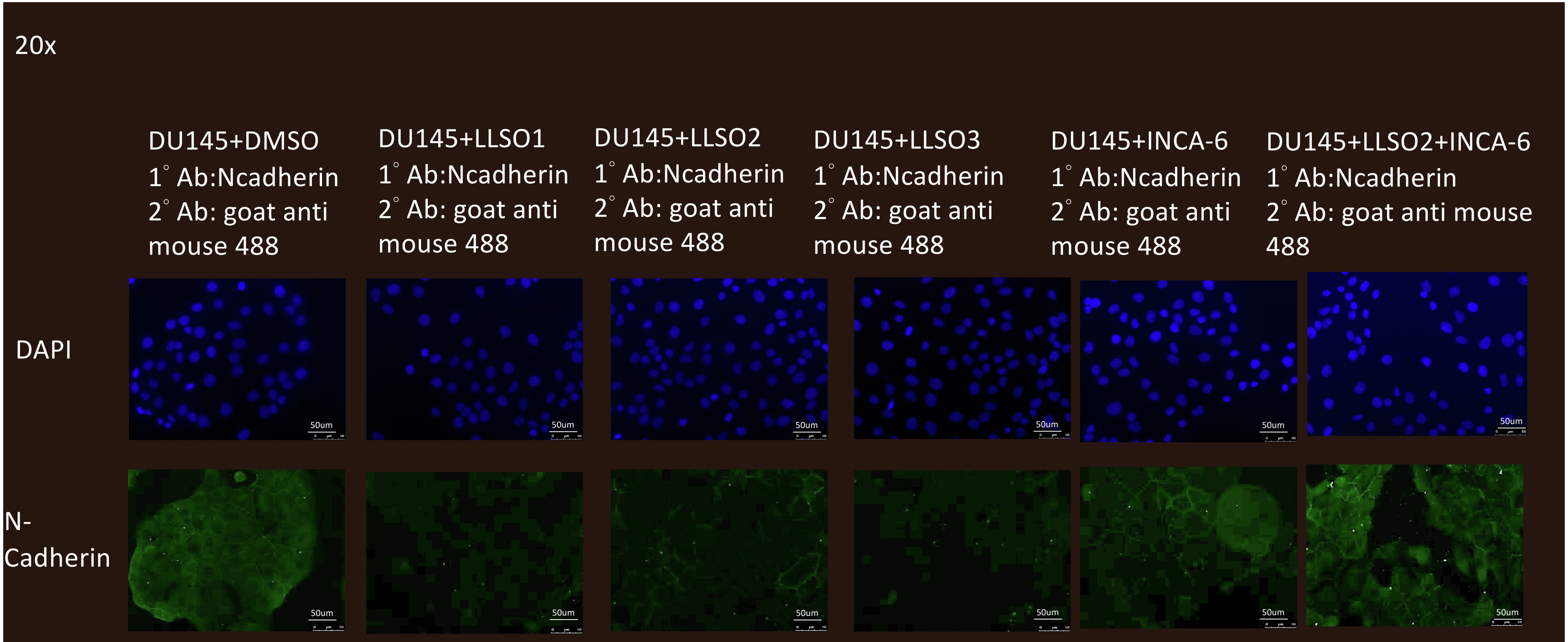

Supplementary Figure 9. Immunofluorescence analysis of DU145 cells treated with LLSO1, LLSO2 or LLSO3 at 10 uM for 48 hours shows a decrease in expression of N-cadherin. When INCA-6 is used or INCA-6 and LLSO2, N-cadherin expression is rescued. Representative examples from at least 10 microscopic fields per experiment, and experiments repeated 3 times Pictures taken at 200 X magnification.

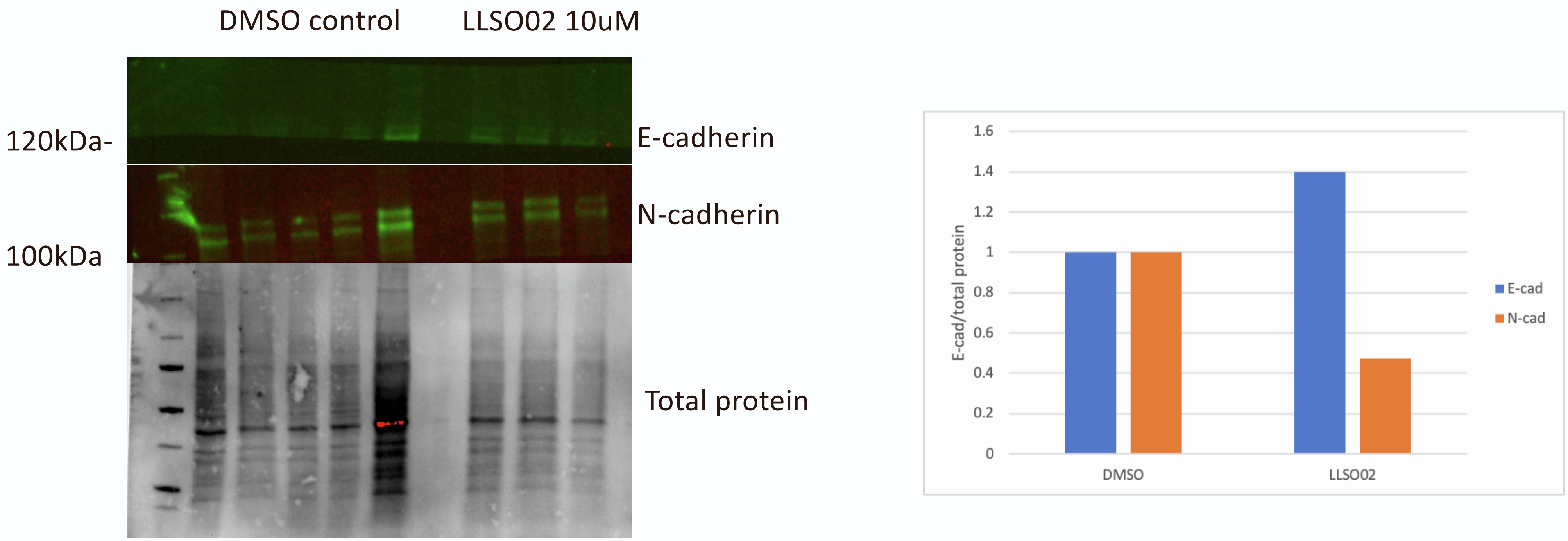

Supplementary Figure 10. LLSO02 induces MET changes in tumour xenografts. Western blot analysis of protein extracts from excised tumours show an increase in E-cadherin and a decrease in N-cadherin, consistent with inducing MET (n=3 replicates, 1 experiment).

LC50 for PC3 treated with LLSO1

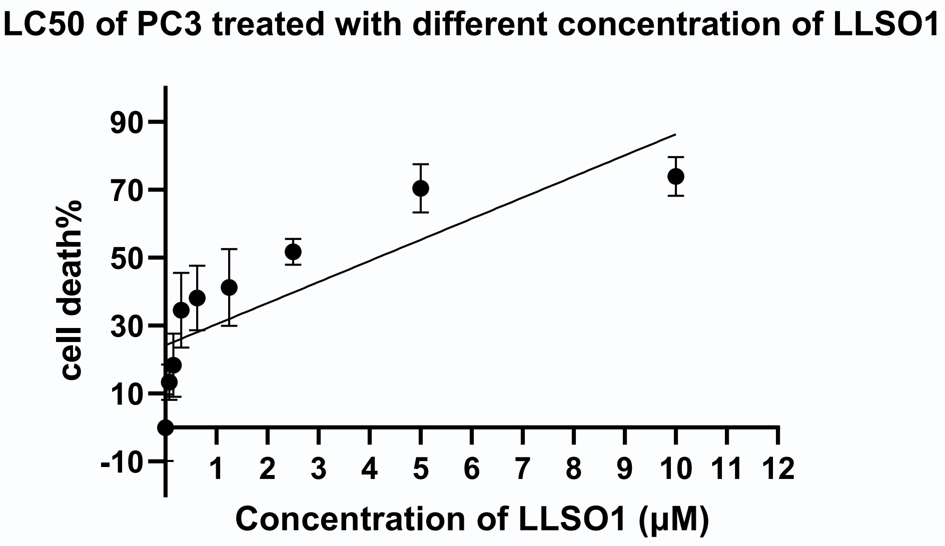

Y = 6.216*X + 24.22

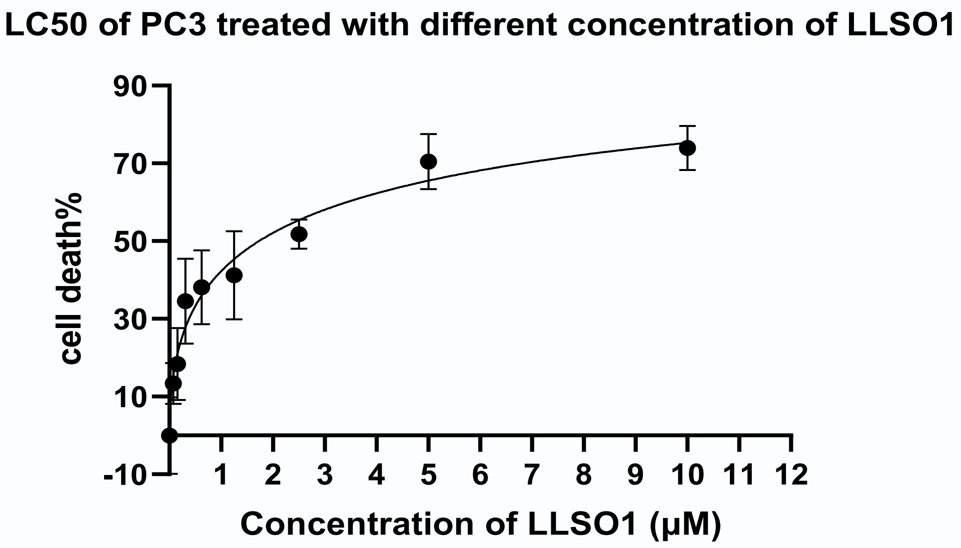

Y = 6.216*X + 24.22

Y = mx + c, Y = 6.216*X + 24.22, m = 6.216, c = 24.22

According to LC50 = [(50-c)/m] formula, LC50 for PC3 treated with LLSO1 is (50-24.22)/6.216 = 4.15μM

Supplementary Figure 11. The LC50 curve of PC3 treated with different concentration of LLSO1. MTT assay was performed following 48 hours pre-treatment of DMSO and different concentration of LLSO1 in PC3 cells. Absorbance rate on MTT assay of PC3 treated with 0.078125μM, 0.15625μM, 0.3125μM, 0.625μM, 1.25μM, 2.5μM, 5μM, 10μM LLSO01, and DMSO (as control) at OD = 590nm. Six replicated within each experiment. The LC50 for PC3 treated with LLSO1 is 4.15μM

Leo Simanjuntak et al 2018 IOP Conf. Ser.: Mater. Sci. Eng. 420 012144

LC50 for PC3 treated with LLSO2

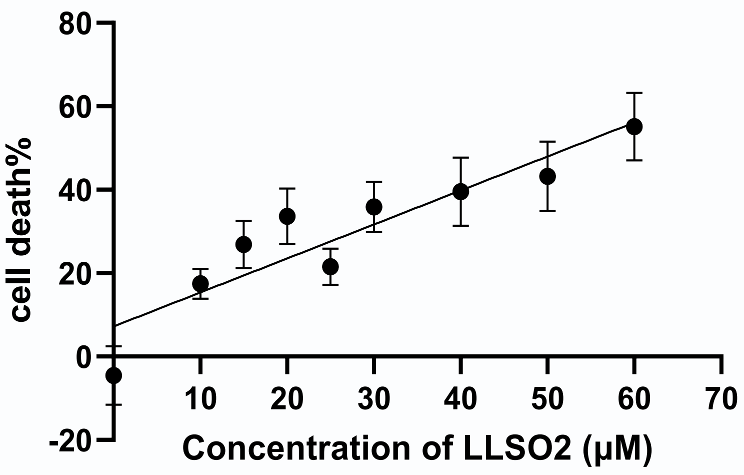

Y = 0.8152*X + 7.220

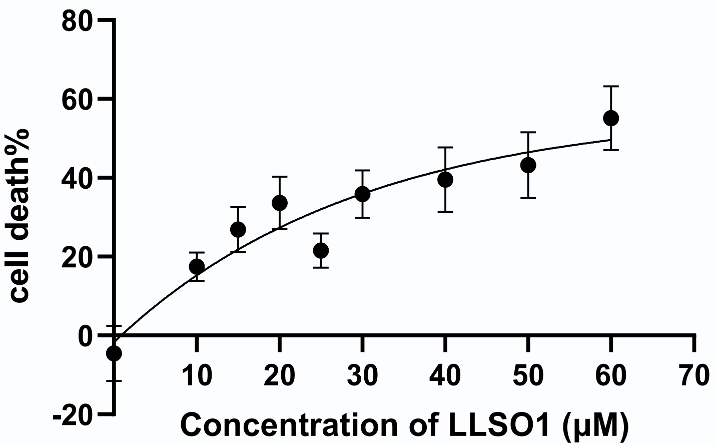

Y = 0.8152*X + 7.22

Y = mx + c,   Y = 0.8152*X + 7.22,   m = 0.8152, c = 7.22

According to LC50 = [(50-c)/m] formula, LC50 for PC3 treated with LLSO1 is (50-7.22)/0.8152 = 52.48μM

Supplementary Figure 12. The LC50 curve of PC3 treated with different concentration of LLSO2. MTT assay was performed following 48 hours pre-treatment of DMSO and different concentration of LLSO2 in PC3 cells. Absorbance rate on MTT assay of PC3 treated with 10μM, 15μM, 20μM, 25μM, 30μM, 40μM, 50μM, 60μM LLSO02, and DMSO (as control) at OD = 590nm. Six replicated within each experiment. The LC50 for PC3 treated with LLSO2 is 52.48μM

LC50 for PC3 treated with LLSO3

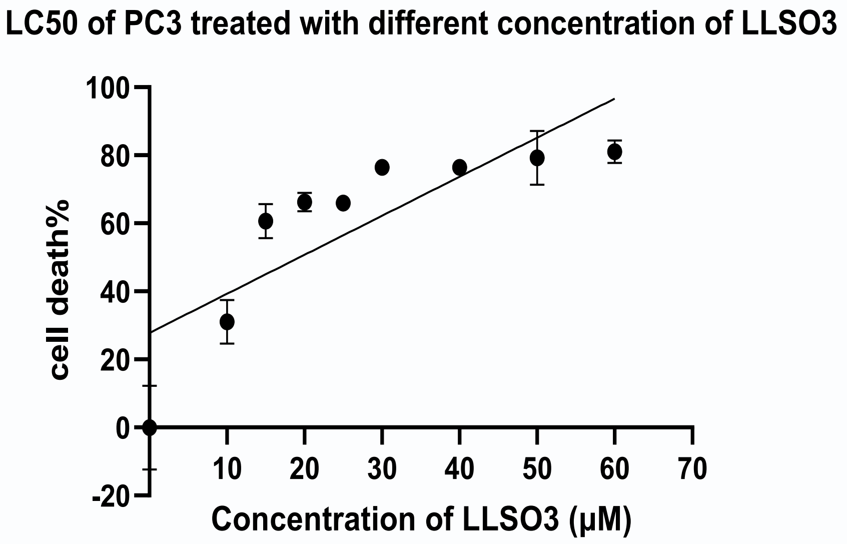

Y = 1.148*X + 27.79

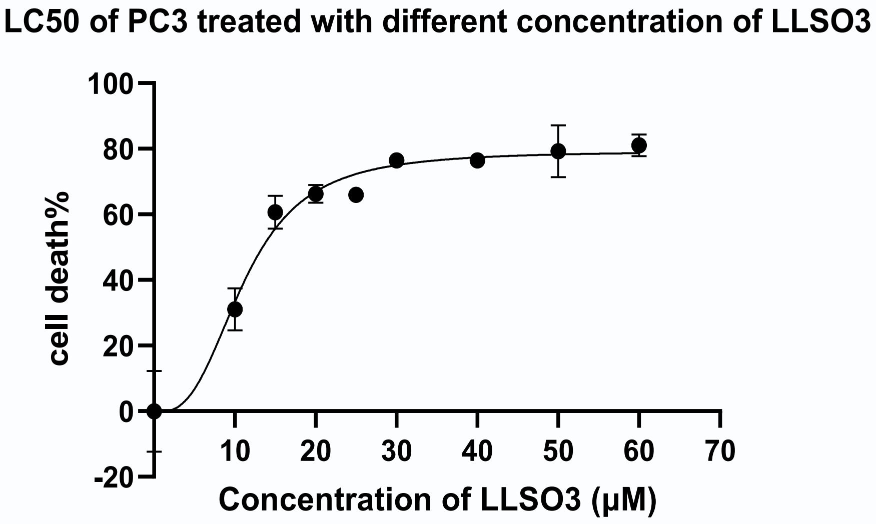

Y = 1.148*X + 27.79

Y = mx + c, Y = 1.148*X + 27.79, m = 1.148, c = 27.79

According to LC50 = [(50-c)/m] formula, LC50 for PC3 treated with LLSO1 is (50-27.79)/1.148 = 19.35μM

Supplementary Figure 13. The LC50 curve of PC3 treated with different concentration of LLSO3. MTT assay was performed following 48 hours pre-treatment of DMSO and different concentration of LLSO3 in PC3 cells. Absorbance rate on MTT assay of PC3 treated with 10μM, 15μM, 20μM, 25μM, 30μM, 40μM, 50μM, 60μM LLSO03, and DMSO (as control) at OD = 590nm. Six replicated within each experiment. The LC50 for PC3 treated with LLSO3 is 19.35μM
