# Supplementary material for: A repositioning screen using an FGFR2 splicing reporter reveals compounds that regulate epithelial-mesenchymal transitions and inhibit growth of prostate cancer xenografts
Source: Mol Ther Methods Clin Dev. 2022 Mar 17;25:147–57. doi: 10.1016/j.omtm.2022.03.005 (PMC8971352; doi:10.1016/j.omtm.2022.03.005)
Supplement: Document S2. Article plus supplemental information [file mmc2.pdf]

Molecular Therapy
Methods & Clinical Development
Original Article

A repositioning screen using an FGFR2 splicing reporter reveals compounds that regulate epithelial-mesenchymal transitions and inhibit growth of prostate cancer xenografts

Ling Li,[1] Jinxia Zheng,[1] Megan Stevens,[1] and Sebastian Oltean[1]

[1]Institute of Biomedical & Clinical Sciences, Medical School, College of Medicine and Health, University of Exeter, St Luke's Campus, Exeter EX1 2LU, UK

Research in the area of hallmarks of cancer has opened the possibility of designing new therapies based on modulating these cancer properties. We present here a screen designed to find chemicals that modulate epithelial-mesenchymal transitions (EMTs) in prostate cancer. For screening, we used a repurposing library and, as a readout, an FGFR2-based splicing reporter, which has been shown previously to be a sensor for EMTs. Various properties of cancer cells were assessed, signaling pathways investigated, and *in vivo* experiments in nude mice xenografts performed. The screen yielded three hit compounds (a T-type Ca channel inhibitor, an L-type Ca channel inhibitor, and an opioid antagonist) that switch FGFR2 splicing and induce an epithelial phenotype in prostate cancer cells. The compounds affected differently various properties of cancer cells, but all of them decreased cell migration, which is in line with modulating EMTs. We further present mechanistic insights into one of the compounds, nemadipine-A. The administration of nemadipine-A intraperitoneally in a nude mouse xenograft model of prostate cancer slowed tumor growth. To conclude, we show that knowledge of the molecular mechanisms that connect alternative splicing and various cancer properties may be used as a platform for drug development.

INTRODUCTION

In the last 20–30 years, there has been a revolution in anticancer treatments involving the rational design of targeted therapies against many molecular targets. While a complete cure of all cancers may never be achieved, the availability of a wide range of targeted therapies against multiple molecules, which may be used in both generalized and personalized therapeutics, will give rise to the possibility of transforming many cancers in chronic diseases, as they are managed more effectively.

Prostate cancer (PCa) is the most commonly diagnosed non-skin cancer and the third most common cause of cancer death in men in the Western world.[1–4] While many men have indolent disease that can be cured with localized therapies, a significant minority of men will relapse and eventually progress to castration-resistant PCa. The majority of lethal PCa is due to spread through castration-resistant metastasis.[5,6] In spite of several novel treatments approved recently, there is still an important need for novel therapeutic targets.

A process that has not yet been targeted therapeutically in PCa is the epithelial-mesenchymal transition (EMT), which is the reversible interchanges between an epithelial phenotype and a mesenchymal one. The EMT occurs in embryonic development and is largely shut down in adult life, with the exception of a few processes such as wound healing; cancer cells re-activate this program and use it to spread and metastasize.[7–10] As is the case with many cancers, there is a clear relationship between the EMT and the progression of PCa, which is demonstrated by the association of EMT regulatory molecule expression with advanced PCa.[11] Furthermore, experiments in mouse models of PCa have shown that the manipulation of EMT regulators results in slowed tumor growth and/or metastasis.[12]

The EMT is regulated at many levels, including alternative splicing (AS); the splice factors ESRPs (epithelial splicing regulatory proteins) and RBFOX1 (RNA binding Fox-1 homolog-1) are the most frequently implicated.[13] ESRPs have been shown to be master regulators of the EMT by coordinating the splicing of more than 200 genes, many of which are involved in the migration properties of cells.[14] In work published recently,[12] we have investigated ESRPs in PCa, showing that both ESRP1 and ESRP2 are overexpressed in PCa and that ectopic expression in PC3 cells is able to slow tumor growth in nude mice xenografts. This indicates that ESRPs do not have redundant functions with other EMT regulators (e.g., transcription factors), and therefore, may represent attractive therapeutic targets. Moreover, ESRP2 is regulated by androgens and is suggested to drive an extensive splicing network in PCa, which has been confirmed recently by another group.[15]

Received 10 March 2021; accepted 13 March 2022;
https://doi.org/10.1016/j.omtm.2022.03.005.

Correspondence: Sebastian Oltean, MD, PhD, Institute of Biomedical & Clinical Sciences, Medical School, College of Medicine and Health, University of Exeter, St Luke's Campus, Exeter, EX1 2LU, UK.
E-mail: s.oltean@exeter.ac.uk

This is an open access article under the CC BY license (http://creativecommons.org/licenses/by/4.0/).

One of the targets of ESRPs is fibroblast growth factor receptor 2 (FGFR2). FGFR2 belongs to the family of tyrosine kinase receptors and it is important in cell survival, proliferation, and differentiation.[16] AS of two mutually exclusive exons (exon 8 or IIIb and exon 9 or IIIc) in the third immunoglobulin-like domain of FGFR2 results in two isoforms that have different ligand-binding specificities.[17] The IIIb isoform is specific to epithelial cells, while the IIIc isoform is expressed in mesenchymal cells; therefore, there is a switching of isoforms during the EMT. An increasing body of literature has implicated the aberrant expression of FGFR2 isoforms in carcinogenesis.[16] A switch from the IIIb to the IIIc isoform has been shown to be involved in the progression of PCa.[17,18] In addition, decreased expression of the IIIb isoform and increased expression of IIIc has been reported in prostate, urothelial, renal, pancreatic, salivary, and colon carcinomas.[19–23]

Given both the presence of the EMT and aberrant AS in aggressive PCa, treatments that are able to modulate EMT states and at the same time correct aberrant AS may be beneficial in both metastatic and non-metastatic, castration-resistant PCa.

We present here a repositioning screen designed to identify chemicals that are able to switch FGFR2 splicing from exon 9 (also called IIIc) to 8 (also called IIIb). Since this splicing switch is also a sensor of the EMT,[24] we show that the hit chemicals modulate the EMT in PCa cells. We also characterize in more detail the mechanism of how one of the compounds works, and present proof-of-principle experiments in nude mice xenografts showing that the administration of such compounds are effective in slowing tumor growth *in vivo*.

RESULTS

A screen using a repositioning library and a splicing reporter reveals three compounds that switch FGFR2 splicing

In a previous report,[24] we described a bichromatic reporter, based on FGFR2 exon IIIc splicing, that functions as a sensor of epithelial and mesenchymal states and transitions. The reporter expresses GFP in mesenchymal cell lines (where exon IIIc is included) and red fluorescent protein (RFP) in epithelial cells (where exon IIIc is skipped). We aimed to use this reporter to screen for compounds that are able to reverse mesenchymal states toward epithelial states.

For ease of translational therapeutic applications, we decided to use a repositioning library, LOPAC from Sigma (St. Louis, MO, USA), that contains 1,280 pharmacologically active drugs. It contains marketed drugs and pharmaceutically relevant structures covering all major target classes (https://www.sigmaaldrich.com/life-science/cell-biology/bioactive-small-molecules/lopac1280-navigator.html).

For the screening cell line, we used HEK293, which has been described previously to exclusively express FGFR2 IIIc inclusion. This allows us to screen from inclusion of IIIc (high GFP/low RFP and corresponding to mesenchymal phenotypes) toward skipping of exon IIIc (low GFP/high RFP and corresponding to epithelial phenotypes).

The design of the screen is shown in Figure 1. In the primary screen, HEK293 cells stably transfected with the bichromatic reporter (pRGIIIc) were plated in the 96-well format and treated with the LOPAC library compounds at 10 μM for 48 h. The fluorescence of each well was read in both RFP and GFP channels on a plate reader and analyzed in comparison with DMSO-treated controls (Figure S1). In this first step, 278 compounds were selected that either increased RFP, decreased GFP, or both. A control screen was needed to eliminate false positives, for example, compounds that fluoresce themselves, compounds that directly affect the red and/or green fluorescent proteins, or compounds that affect the reporter RNA or protein stability and/or translation. For this purpose, control reporters have been designed that lack the intronic parts of the initial reporter and mimic the two possible spliced transcripts of the initial reporter (Figure 1, step 2, control screen). Two additional screens were performed with these control reporters, which was used to eliminate false positives. For example, if in the primary screen a certain compound would show a significant increase in RFP but this would be shown in the secondary/elimination screen as well, then this compound would be deemed a false positive (for more details and examples of the elimination procedure of false positives, see Figure S2). After this step, 124 compounds remained that were validated for the effect on FGFR2 splicing by RT-PCR and restriction digestion analysis. While several compounds have been validated at the RT-PCR level, we have chosen to pursue further three compounds that induced the greatest increase in the inclusion of IIIb (Figure 2). These compounds were called LLSOs (after the main authors' initials): LLSO01 (NNC-55-0396 dihydrochloride) is a structural analog of mibefradil, which is a highly selective T-type calcium channel blocker; LLSO02 (nemadipine-A) is an analog of 1,4-dihydropyridine, which is an L-type calcium channel α1-subunit antagonist; and LLSO03 (naltrexone hydrochloride) is an opioid antagonist, which is a synthetic congener of oxymorphone with no opioid agonist properties. While the initial validation was made in HEK293 cells, we have also demonstrated by RT-PCR that the LLSO compounds are changing FGFR2 splicing in PC3 prostate cancer cells (Figure S3).

LLSOs induce the expression of E-cadherin in PC3 cells

The purpose of the screen was to find compounds that modulate the EMT, since a switch in FGFR2 from exon IIIc to IIIb has been linked previously to mesenchymal-to-epithelial transitions (METs). Therefore, we wanted to determine whether LLSOs modulate the expression of EMT markers.

PC3 cells were treated with individual LLSOs for 48 h at 10 μM, and the expression of E-cadherin was assessed. On western blot analysis, a moderate increase in E-cadherin levels was observed (Figure 3A). However, when analyzed by immunofluorescence, the induction of E-cadherin expression is much more evident; it is not only increased in expression but also shows an increase in its junctional localization (Figure 3B).

Another established marker of the EMT, cytokeratin, has also shown a change in expression in the expected direction for MET, albeit not as

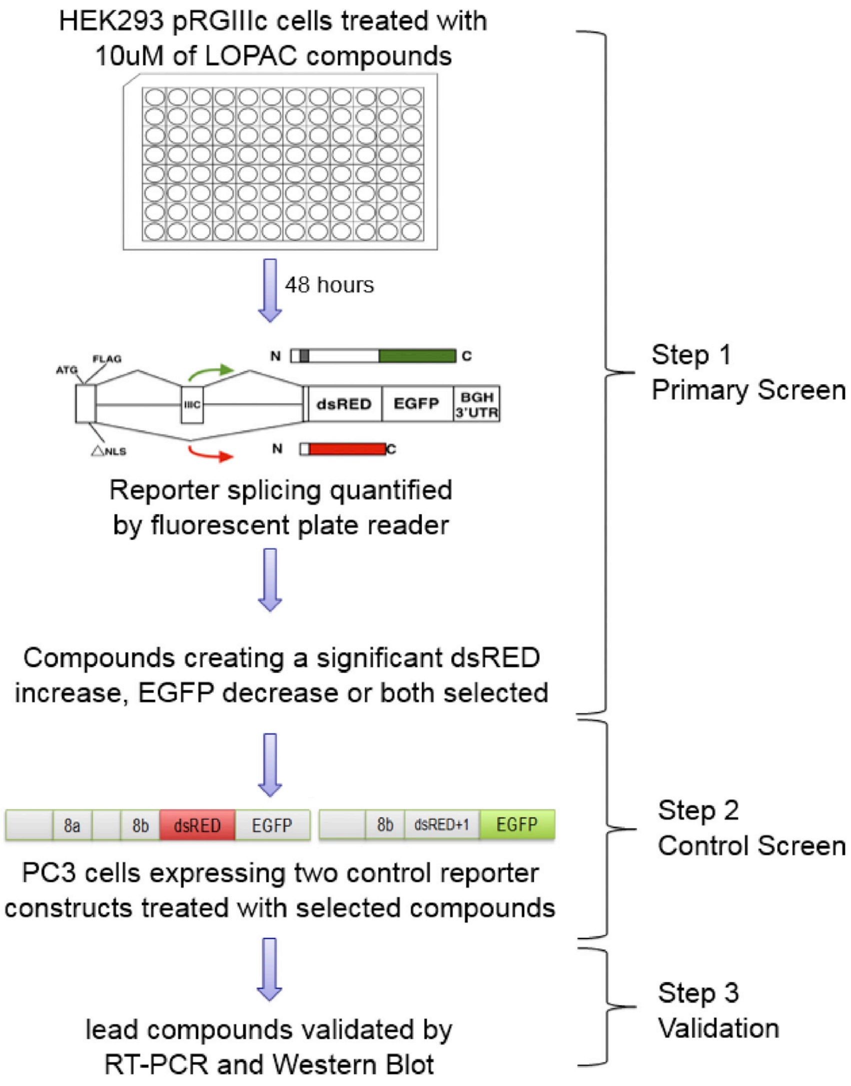

Figure 1. Schematics of the screen using the LOPAC library and the FGFR2 splicing reporter

HEK293 cells transfected with the fluorescent FGFR2 splicing reporter were treated with the LOPAC compound library and reporter splicing measured using a fluorescent plate reader (primary screen, step 1). Selected compounds were used to eliminate false positives by repeating the screen with control reporter constructs that cannot be spliced (control screen, step 2). Lead compounds were validated using RT-PCR and western blot (step 3).

evident as E-cadherin (Figure S4). Finally, N-cadherin, a mesenchymal marker, decreases its expression in PC3 cells when treated with LLSOs (Figure S5).

LLSOs affect growth, migration, and proliferation of PC3 cells differently

To characterize the effect of LLSOs on PC3 cell properties, we performed several assays *in vitro*. Cell counting showed very different responses. At 10 μM, LLSO01 was toxic and killed a majority of the cells; LLSO02 had no effect on cell growth; and LLSO03 slowed the growth rate (Figure 4A). Because of the toxicity, LLSO01 was used at lower concentrations (5 μM) in later experiments. As expected for compounds that modulate the EMT, all LLSOs inhibited the migration of cells, as assessed in a Boyden chamber assay (Figure 4B). Finally, the proliferation rate was assessed using the MTT assay; LLSO01 significantly inhibited proliferation at 5 μM, while no effect was observed for LLSO02 and 03 at 10 μM (Figure 4C). Lethal concentration 50 (LC_{50}) analysis for all 3 compounds (concentration at which they are lethal to 50% of cells) is presented in Figures S11–S13.

LLSO02-modulated increase in E-cadherin expression involves nuclear factor of activated T cells (NFAT) and cyclic AMP response element-binding protein (CREB) transcription factors

We further selected LLSO02 to determine the mechanism through which it signals to increase E-cadherin expression. Theoretically, when investigating compounds that may be repurposed, their new effect can be through the well-established canonical mechanism or a completely new one. LLSO02, nemadipine-A, is an L-type calcium channel inhibitor; L-type channels are part of the voltage-gated calcium channels family (VGCC). Ca channels have been reported to be expressed both in the normal prostate and in PCa cells, including PC3 cells.[25–27] Indeed, western blot analysis shows that L-type Ca channels are expressed in PC3 cells (Figure S6). Therefore, we investigated whether the canonical signaling pathway of L-type Ca channels was involved in the effect of LLSO02. Knowing the molecular heterogeneity of PCa, we probed this mechanism in several cell lines: PC3, LNCaP, and DU145.

Activation of VGCCs results in an increase in the intracellular Ca concentration and activation of calmodulin, calcineurin, and the transcription factors NFAT and CREB (Figure 5A).[27] Therefore, we tested whether NFAT and/or CREB are involved by using INCA-6 as an NFAT inhibitor[28,29] and 666-15 as a CREB inhibitor.[30] In PC3 cells treated individually with these inhibitors, E-cadherin expression was increased similar to LLSO02 treatment (Figure 5B), suggesting that these pathways may be involved. This was reproduced in the LNCaP cell line (Figure S7). However, in DU145, while the CREB inhibitor reproduced the E-cadherin activation, the NFAT inhibitor did not (Figure 5C). Indeed, when DU145 cells were treated with both LLSO02 and the NFAT inhibitor combined, the activation of

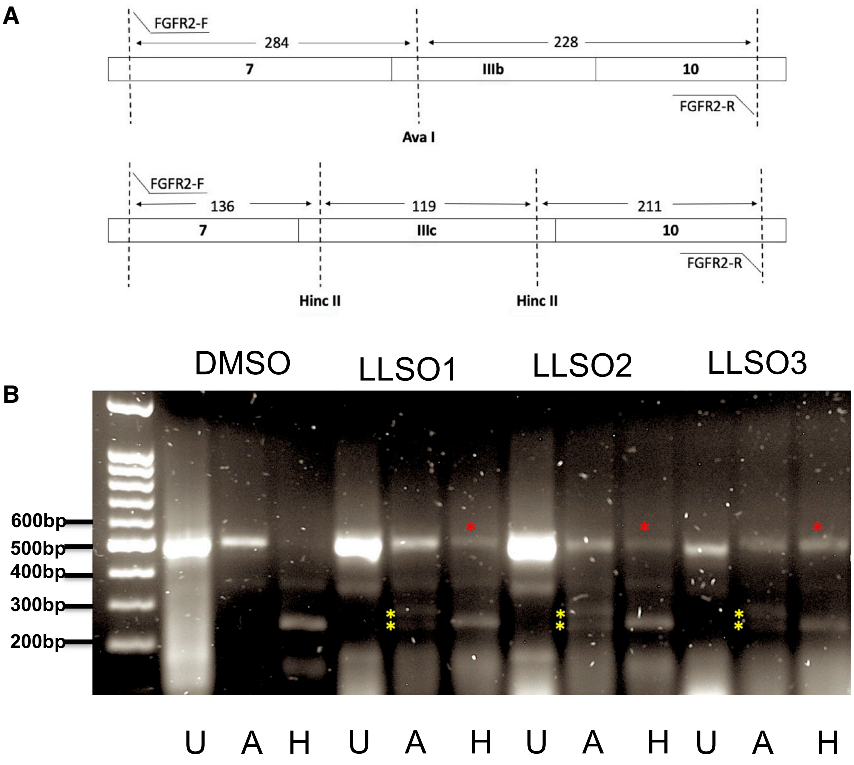

Figure 2. All 3 LLSO compounds switch FGFR2 splicing from complete inclusion of exon IIIc and no IIIb to a mixture of IIIb and IIIc inclusion

(A) Schematics of the assay to assess presence of exons IIIb or IIIc in FGFR2 transcripts. RT-PCR with flanking primers in exons 7 and 10 gives a PCR of similar size, regardless of IIIc or IIIb being present. To distinguish the exons, PCR products are differentially digested with AvaI (cuts only IIIb) and Hinc II (cuts only IIIc). (B) HEK293 cells treated with DMSO, LLSO01 5 μM, LLSO02 10 μM, and LLSO03 10 μM for 48 h. U, FGFR2 PCR products without restriction enzyme treatment; A, FGFR2 PCR products with restriction enzyme treatment by *Ava* I, which only cuts the FGFR2 IIIb isoform; H, FGFR2 PCR products with restriction enzyme treatment by *Hinc* II, which only cuts the FGFR2 IIIc isoform. In the DMSO treatment, there is nothing left undigested in the H column, since all of it is exon IIIc. In the A columns of the LLSO treatments, one can clearly see the expected digestion products for IIIb (yellow stars). The band in the H columns of the LLSOs treatments that remains undigested is the induced IIIb exon (red stars). The gel shown is representative of a minimum of 3 repeats.

E-cadherin by LLSO02 was abrogated, suggesting that in this cell line, the signaling pathway of LLSO02 involves other mechanisms as well. As expected, staining for N-cadherin in the same cells and with the same treatments shows the opposite movement in expression as compared to E-cadherin (see Figures S8 and S9).

Systemic administration of LLSO02 decreases tumor growth in subcutaneous PC3 xenografts in nude mice

We wanted to further explore whether LLSOs also affect tumor growth *in vivo*. Since the property we were investigating is the potential inhibition of the EMT, we decided to test LLSO2, which did not have any effect on cell growth or proliferation (Figures 4A and 4C). We designed a therapeutic proof-of-principle experiment in which 1 million PC3 cells were injected subcutaneously into the flank of nude mice. Tumor sizes were measured twice weekly using a caliper; when the tumors reached 3 × 3 mm in diameter, treatments were started with either vehicle or LLSO02 at 2.936 mg/kg body weight (corresponding to approximately 10 μM 3 times weekly. As seen in Figure 6, there is a clear decrease in tumor growth in mice treated with LLSO02.

Protein extracts prepared from the excised tumors were analyzed for the expression of EMT markers. Indeed, as expected, there was an increase in LLSO02-treated tumors in E-cadherin expression and a decrease in N-cadherin expression, as expected for an MET (Figure S10).

DISCUSSION

The hallmarks of cancer provide a framework for identifying key molecular regulators that can be targeted to obtain new anticancer therapeutics. We have focused on one such hallmark, the EMT. In a quest to find the modulators of the EMT, we performed a repositioning screen using an FGFR2-based splicing reporter and obtained three compounds that are able to (1) induce the expression of epithelial markers in PCa cells and (2) switch FGFR2 splicing from exon IIIc (9) inclusion, which is associates with aggressive cancer behavior, to exon IIIb (8), which is therapeutically beneficial. The three compounds are described in detail below:

- LLSO01: NNC-55-0396 dihydrochloride is a structural analog of mibefradil, an inhibitor of both T-type and L-type Ca channels, which has been used for some time in the clinic to treat hypertension and chronic angina pectoris. LLSO01 has been developed as a more selective inhibitor that acts mainly on T-type Ca channels.[31,32]
- LLSO02: Nemadipine A is an analog of 1,4-dihydropyridine (DHP), which is an L-type Ca channel α1-subunit antagonist. Drugs in this class are normally used to treat hypertension, angina, and heart attacks.[33]
- LLSO03: Naltrexone hydrochloride is an opioid antagonist, a synthetic congener of oxymorphone with no opioid agonist properties. It inhibits the effect of narcotics on the central nervous system, and is commonly used as an analgesic to treat moderate to severe pain.[34]

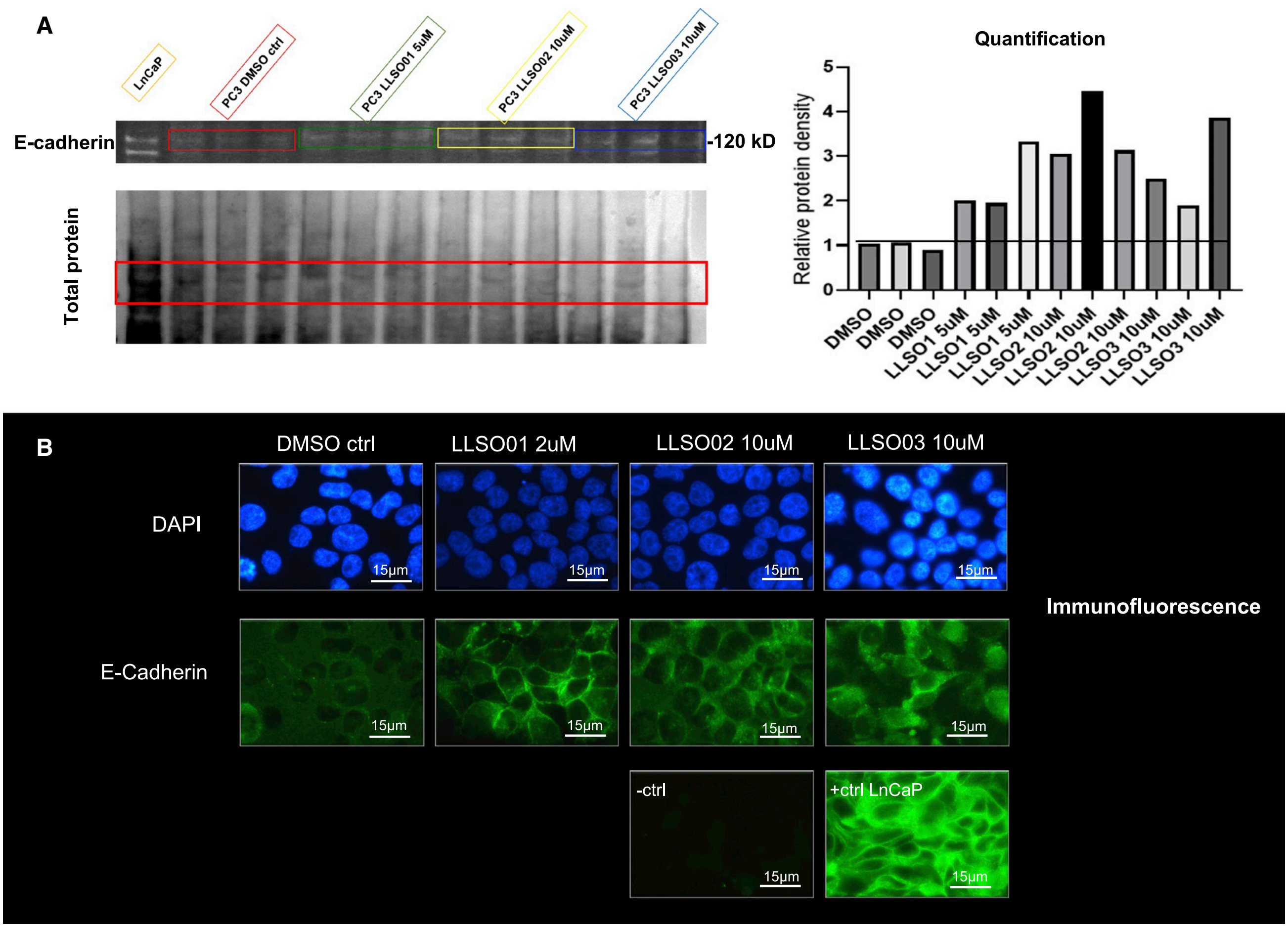

Figure 3. LLSOs change both the expression and localization of E-cadherin in PC3 cells

(A) Western blot for E-cadherin expression was performed with proteins extracted following 48-h treatments with DMSO and LLSOs in PC3 cells. Quantification of the E-cadherin blot is shown in the histogram on the right. All 3 chemicals showed an increase in the E-cadherin expression level. Normalization of the E-cadherin signal is done with the GelEZ system (Bio-Rad), which quantifies total protein on the transfer membrane (left lower panel). The experiment was repeated 3 times. (B) Immunofluorescence analysis was performed following 48-h treatments with DMSO and LLSOs in PC3 cells. PC3 cells stained with mouse IgG were used as a negative control and LNCaP cells were used as a positive control. All 3 chemicals induce an increase in E-cadherin expression levels as well as a more junctional localization. Representative examples from at least 10 microscopic fields per experiment, and experiments repeated 3 times. Photomicrographs were taken at 1,000× magnification; scale bar is 15 µm.

Ca channels have been previously implicated in cancer and ideas have been described as to how to use them for novel therapeutic development, although none of their inhibitors have yet made it to the clinic.[35,36] In particular, for PCa, epidemiological studies have shown that voltage-gated Ca channels blockers (a class to which L- and T-type Ca channels belong) reduce the associated risk of PCa and reduce tumor aggression.[37] In addition, some Ca channels, including transient receptor potential (TRP)V2 and TRPM7, have been implicated in regulating the migration and invasion of PCa cells as well as the regulation of E-cadherin expression.[38] There is also a connection between Ca channels and EMT regulation. For instance, a recent paper described the relationship between the transcription factor ZEB1, a master regulator of the EMT, and Ca channels.[39]

Opioid receptors and signaling have also been implicated in cancer progression. In particular, for PCa, it has been shown that some opioid receptors may stimulate cancer growth through the activation of angiogenesis and that high receptor expression is associated with decreased progression-free survival and overall survival.[40]

While the pharmaceutical classes to which the three LLSO compounds belong have been previously implicated in cancer therapeutics, to the best of our knowledge, none of the LLSOs have been described as possible treatments for PCa. In line with our findings, mibefradil, to which LLSO01 is structurally related, has been shown to reduce the viability of PCa cells in culture.[41] The only previously reported connection to cancer for LLSO02 is a resultant increase in apoptosis in lung cancer cells.[42] LLSO03 has been reported to have

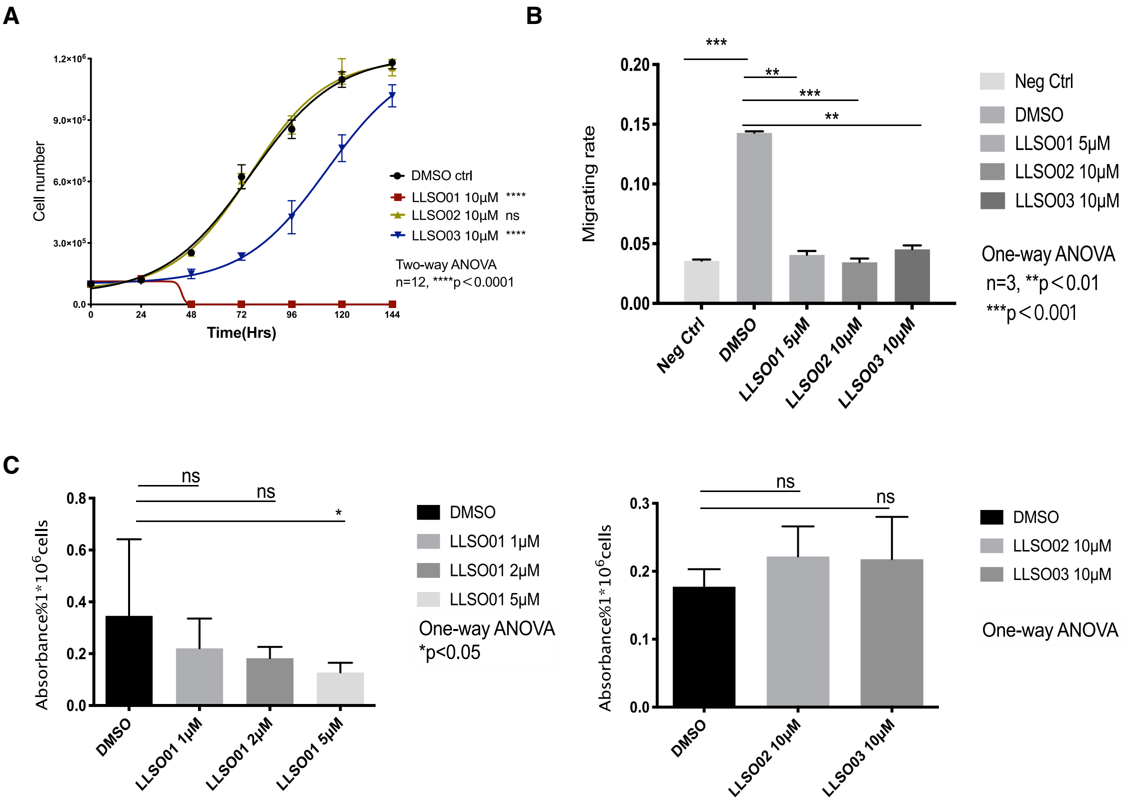

Figure 4. LLSOs affect PC3 cell properties in different ways

(A) The growth curve of LLSOs (all 10 μM) and DMSO-treated PC3 cells. LLSO01 is toxic to PC3 cells and LLSO02 shows no significantly effect on cell growth, while LLSO03 shows a significant decrease in cell growth. Medium with drugs was refreshed every 48 h after seeding. Cells were counted every 24 h after seeding in the plate. All of the treatments had 12 repeats. n = 12, ***$p < 0.001$ by 2-way ANOVA. (B) The Boyden chamber assay was performed following a 48-h pre-treatment of PC3 cells with DMSO or LLSOs. PC3 cells were cultured overnight in reduced medium with 2% FBS to starve the cells, and PC3 cells without starving were used as negative controls. The graph shows a normalized migration rate in the Boyden chamber assay of LLSO01 5 μM, LLSO02 10 μM-treated, LLSO03 10 μM-treated PC3 cells, and DMSO (as control)-treated PC3 cells. n = 3; the data were analyzed using 1-way ANOVA. (C) MTT assay was performed following 48 h pre-treatment of PC3 cells with DMSO and LLSOs. Left panel: Absorbance rate on MTT assay of PC3 treated with 1 μM, 2 μM, and 5 μM LLSO01 and DMSO (as control) PC3 cells. Right panel: Absorbance rate on MTT assay of PC3 treated with LLSO02 10 μM, LLSO03 10 μM, and DMSO (as control) PC3 cells. n = 3; the data were analyzed by 1-way ANOVA. ns, not significant.

antitumoral activity;[43] interestingly, a recent report showed that LLSO03 inhibits EMT in cervical cancer cells.[44]

Alternative splicing has been shown to be closely connected to cancer development; there are many reports of aberrant splice isoforms expressed in cancers that maintain oncogenic properties, as well as on splice factors that are oncogenes.[45] Indeed, FGFR2 is such an example. As mentioned above, the isoform containing exon IIIc (or 9) is associated with aggressive cancers and IIIb (or 8) with more favorable outcomes. Moreover, in preclinical studies, it has been shown that forced overexpression of the exon IIIb-containing isoform in PCa cells that express IIIc is therapeutically beneficial,[46,47] suggesting the validity of a therapeutic approach to switch isoforms.

The connection between PCa and aberrant AS is not limited to FGFR2; recent papers have clearly highlighted a major role played by aberrant AS in PCa with many splice isoforms involved as well as splice factors or components of the spliceosome.[48–50] Targeting the mesenchymal phenotype may be a way to eliminate the existing metastatic cancer cells. Some studies have targeted classic mesenchymal proteins such as vimentin to inhibit metastasis and decrease resistance to chemotherapy, with some initial success.[51] For example, withaferin-A is a bioactive compound extracted from *Withania somnifera* that has been shown to promote the degradation of vimentin. Studies in soft tissue sarcoma cells have shown that withaferin-A induces apoptosis, while compared to cells expressing normal levels of vimentin, malignant cell lines with higher levels of vimentin are more sensitive to withaferin-A.[52] A study from another group showed that monoclonal antibodies targeting N-cadherin inhibit prostate cancer growth and reduce invasion, metastasis, and castration resistance.[53]

In conclusion, we have shown that LLSO compounds affect cell growth and proliferation, although in different ways,. Furthermore, all of them inhibit the migration of PCa cells, which is expected for EMT inhibitors. We also provide proof-of-principle experiments

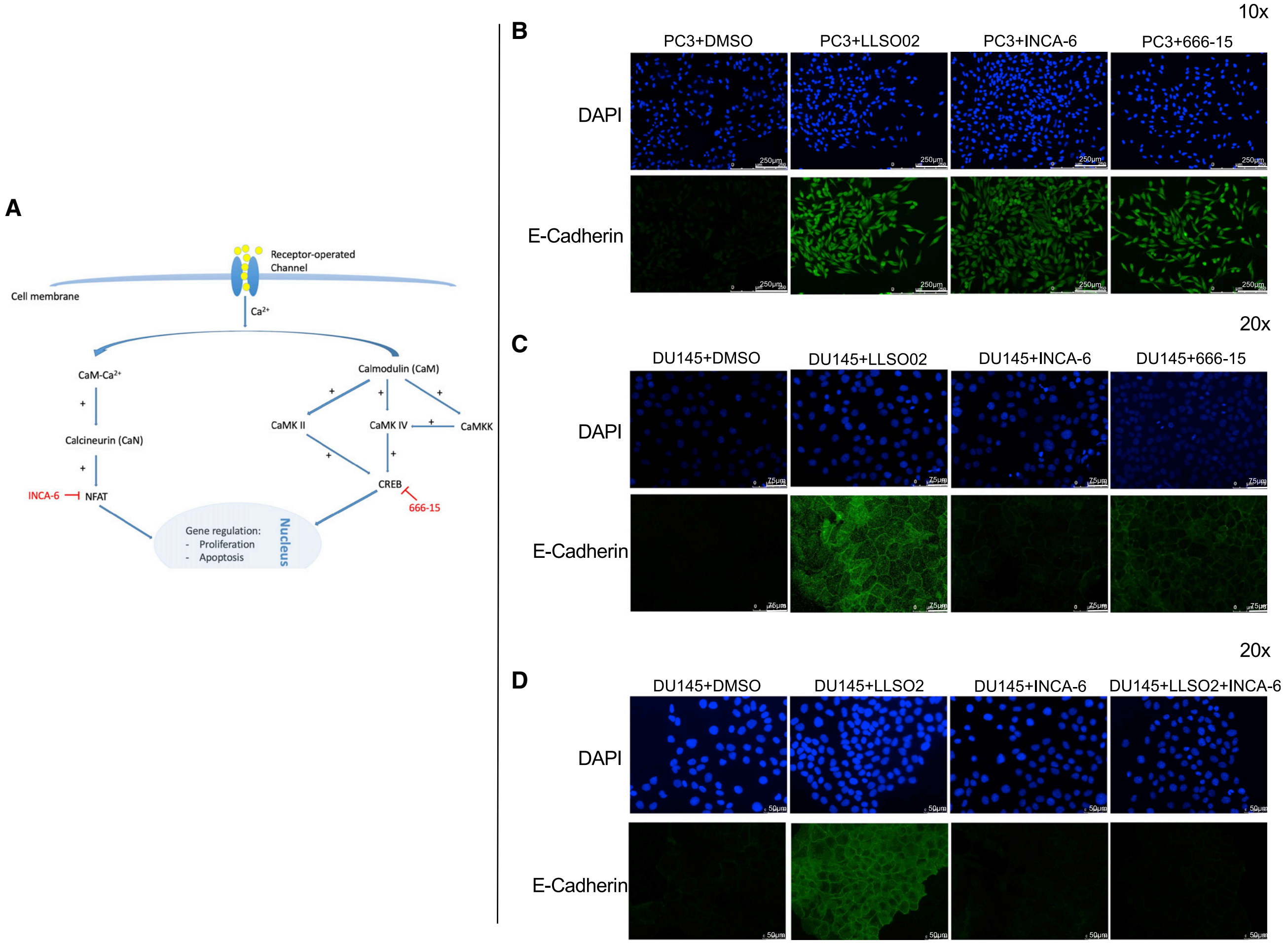

Figure 5. LLSO02 uses NFAT and/or CREB to signal to increase E-cadherin expression in PC3 cells; in DU145 cells additional mechanisms may be in play
(A) Hypothetical signaling pathway for LLSO2. (B) Images of PC3 cells treated separately with 10 μM of LLSO2, 10 μM of INCA-6, and 10 μM of 666-15; scale bar, 250 μm. (C) Images of DU145 cells treated separately with 10 μM of LLSO2, 10 μM of INCA-6, and 10 μM of 666-15; scale bar, 75 μm. (D) Images of DU145 cells treated separately with 10 μM of LLSO2, 10 μM of INCA-6, and the combination of LLSO2 and INCA-6; scale bar, 50 μm. All of the images were taken at 20× magnification. The images are representative for replicates (3 individual experiments, 3 wells for each treatment, and a minimum of 3 microscopic fields surveyed in each well).

showing that one of the compounds is able to inhibit tumor growth *in vivo*, supporting the idea that they may be developed as anticancer therapeutics. To note, for EMT inhibitors, off-target effects on normal cells are less of a concern than with other treatments, as EMT is largely shut down in adult life.

MATERIALS AND METHODS

Cell lines

HEK293 (embryonic kidney cells), PC3, and DU145 (human prostate cancer) cells were obtained from Microvascular Research Laboratories (MVRL), University of Bristol. LNCaP human prostate cancer cells were purchased from the American Type Culture Collection (Manassas, VA, USA). HEK293 cells were grown and cultured in DMEM medium (Sigma) supplemented with 10% fetal bovine serum (Gibco, Waltham, MA, USA) and 1% penicillin-streptomycin (10,000 U/mL penicillin, 10,000 μg/mL streptomycin). PC3 and LNCaP cells were grown and cultured in RPMI 1640 medium (Sigma) supplemented with 10% fetal bovine serum (Gibco) and 1% penicillin-streptomycin (10,000 U/mL penicillin, 10,000 μg/mL streptomycin). All of the cells were grown in a 5% CO_2 atmosphere at 37°C.

Splicing reporter screen

HEK293 cells stably transfected with the splicing reporter pRGIIIc (FGFR2 exon IIIc cloned into the RG6 minigene)[24] were used as reporter cells. We seeded 10,000 cells/well in 96-well plates, which were incubated at 37°C with 5% CO_2. The next day, the cells were treated with compounds from the LOPAC Library (Sigma) at 10 μM for 48 h. Fluorescence intensity was read with a VICTOR plate reader. The data were analyzed using a one-way ANOVA with GraphPad Prism (GraphPad, San Jose, CA, USA). Selected chemicals from the primary screen underwent a control screen following the same protocol but using reporters that do not splice—PC3 DSS

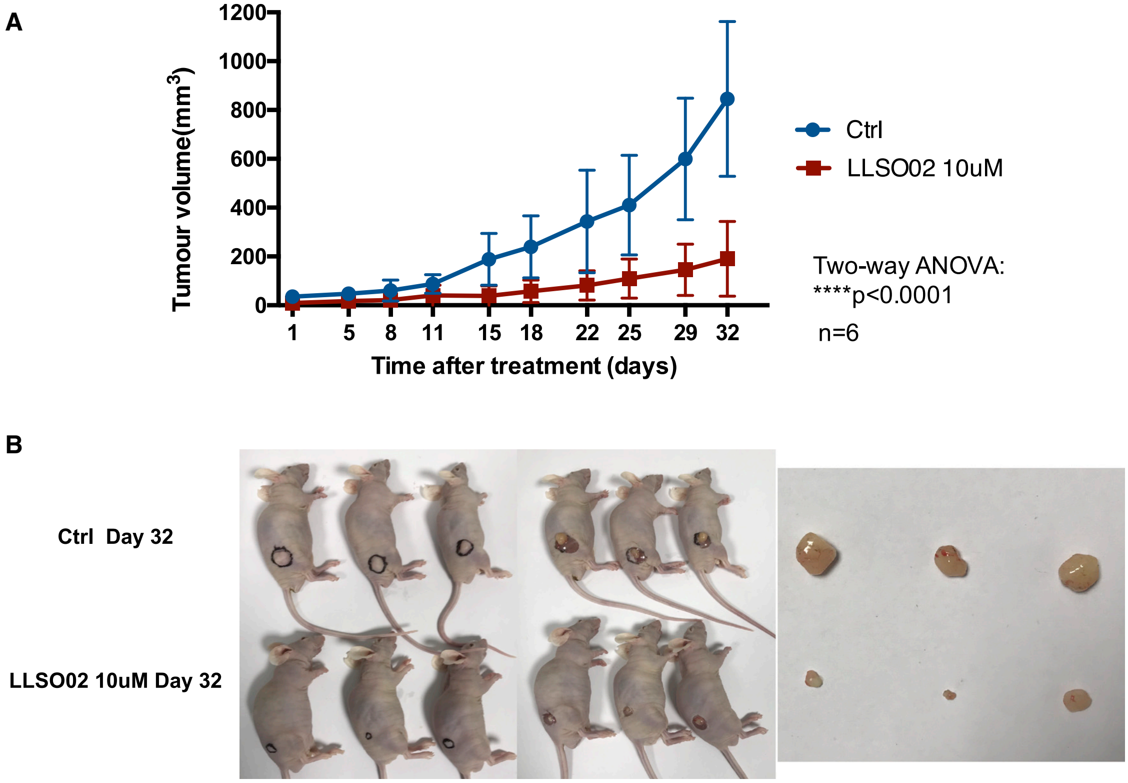

Figure 6. LLSO02 decreases tumor growth when administered systemically in nude mice with PC3 subcutaneous xenografts

(A) Quantitation of the tumor volumes in control and LLSO02-treated mice. (B) Examples of tumor growth in both mice groups. Left: Mice with tumors; tumors outlined in black; right: dissected tumors.

(pRG8ab distal splice site control reporter, GFP) cells and PC3 PSS (pRG8ab proximal splice site control reporter, RFP) cells—to eliminate false positives.

RT-PCR and restriction digestion

HEK293 cells pre-treated with DMSO, LLSO01 (5 μM), LLSO02 (10 μM), and LLSO03 (10 μM) for 48 h were harvested and total RNA extracted using TRI-reagent (Invitrogen, Carlsbad, CA, USA), according to the manufacturer's instructions. RNA was treated with RQ1 Rnase-Free DNase (Promega, Madison, WI, USA) to prevent genomic DNA contamination and cDNA was generated by the reverse transcription of 2 μg of total RNA using M-MLV Reverse Transcriptase (Promega) in accordance with the manufacturer's instructions. PCR was performed in triplicate on cDNA using PCR Master Mix (Promega). PCR products were validated by gel electrophoresis (2% agarose gel) at 90 V for 90 min. Both cDNA and RNA ([RT$^-$] control) were subjected to PCR for FGFR2 IIIb/IIIc expression with specific primers. PCR amplification was initiated at 95°C for 5 min, 36 cycles at 95°C for 30 s, 55°C for 30 s, and 72°C for 30 s, followed by final extension at 65°C for 10 min. The PCR products were digested with two restriction digestion enzymes, AvaI and HincII, to distinguish the IIIb and IIIc isoforms. Each treatment had three repeats.

Immunoblot analysis

Cells were lysed with a RIPA lysis buffer containing 1% PMSF solution, 1% sodium orthovanadate solution, and 1%–2% protease inhibitor cocktail solution on ice. Proteins were separated by SDS-PAGE on TGX stain-free gels (Bio-Rad, Hercules, CA, USA) and activated with the ImageLab Software (Bio-Rad). Next, proteins underwent electrophoretic transfer onto polyvinylidene difluoride membranes, with the total protein on the membrane then able to be quantified with the ImageLab software. Membranes were blocked with 5% BSA in 50 mm Tris-HCl (pH 7.4), 150 mm NaCl, and 0.1% Tween 20. The following primary antibodies were used: anti-vimentin (BD Biosciences, San Jose, CA, USA), anti-N-cadherin (Abcam, Cambridge, UK), anti-cytokeratin (Sigma), and anti-E-cadherin (BD Biosciences). The blots were incubated with the appropriate primary antibody overnight at 4°C. After incubation with a fluorescent secondary antibody (Li-Cor) for 1 h, the proteins were visualized using the Li-Cor Imager.

Immunofluorescence

Cells were seeded on 17 × 17-mm microscope coverslips in 12-well plates at 300,000 cells per well, and treated with DMSO, LLSO01 (5 μM), LLSO02 (10 μM), and LLSO03 (10 μM) for 48 h. Cells were then fixed in 4% paraformaldehyde for 10 min, and permeabilized for 10 min in 0.3% Triton X-100 in PBS. After blocking

with 1% BSA and 5% normal goat serum in PBS for 15 min, cells were incubated overnight at 4°C with anti-E-cadherin primary monoclonal antibody (BD Biosciences) diluted in 1% BSA in PBS. The cells were then incubated with Alexa Fluor 488-labeled goat anti-mouse immunoglobulin G (IgG) as a secondary antibody for 1 h and then with DAPI for 15 min at room temperature. Images were acquired with a Leica fluorescence microscope and analyzed with Leica X imaging software. The cells treated with inhibitors underwent immunofluorescence using the same protocol.

Cell growth curve

PC3 cells were seeded at 100,000 cells per well in a 12-well plate. The day following seeding, cells were treated with DMSO or LLSOs. Cells were counted every 24 h after seeding. All of the data were calculated and analyzed with GraphPad Prism and statistical differences assessed with a two-way ANOVA. All of the treatments had 12 repeats.

Cell migration assay: Boyden chamber assay

Cells were pre-treated with DMSO, LLSO01 (5 μM), LLSO02 (10 μM), and LLSO03 (10 μM) for 48 h, and then serum starved overnight and seeded in 24-well 8.0-μm pore size culture inserts (Millipore, Burlington, MA, USA) at 150,000 cells per well. Complete medium with 10% fetal bovine serum (FBS) was placed in the well outside the inserts. After incubating at 37°C for 24 h, cells that had migrated through the membrane were stained with DAPI and then counted from photomicrographs. The data were analyzed by Prism using a one-way ANOVA. All of the treatments had four repeats.

Proliferation assay: MTT assay

An MTT assay was performed using the Vybrant MTT Cell Proliferation Assay Kit (Invitrogen) per the manufacturer's protocol. PC3 cells were seeded in a 96-well plate at 10,000 cells per well after pre-treating with either DMSO or one of the three compounds at different doses (LLSOs) for 48 h. Three repeats were applied for each compound. Absorbance at 570 nm of each well was measured with an absorbance microplate reader. The data were analyzed by Prism using a one-way ANOVA.

Tumor xenografts

All of the experiments were conducted in accordance with UK legislation and with local ethics committee approval (University of Exeter AWERB). One million PC3 cells were injected subcutaneously into the right flank of male CD-1 nude mice (Charles River Laboratories, Wilmington, MA, USA). Tumors were measured with a caliper twice weekly and the tumor volume was calculated according to the formula [(length + width)/2] × length × width. DMSO or 10 μM LLSO02 was injected intraperitoneally twice weekly once the tumor size reached 3 × 3 mm. There were six mice per treatment group. Mice were culled by cervical dislocation (Schedule 1) when the control tumor sizes reached the allowed endpoint (one diameter 12 mm) and tumors were dissected. Each tumor was imaged and weighed before flash freezing in liquid nitrogen for further analysis.

Statistical analyses

All of the statistical analyses were performed using GraphPad Prism 7. A p value <0.05 was deemed statistically significant.

SUPPLEMENTAL INFORMATION

Supplemental information can be found online at https://doi.org/10.1016/j.omtm.2022.03.005.

ACKNOWLEDGMENTS

This work has been funded by a China Scholarship Council doctoral fellowship to L.L. and BBSRC grant BB/J007293/2 to S.O.

AUTHOR CONTRIBUTIONS

L.L. designed the experiments, performed the experiments, analyzed the data, and edited the manuscript. J.Z. performed experiments and analyzed the data and edited the manuscript. M.S. performed the experiments and analyzed the data and edited the manuscript. S.O. designed the research plan, supervised the study, wrote the first draft of the manuscript, and edited the manuscript.

DECLARATION OF INTERESTS

The authors declare no competing interests.

REFERENCES

1. Siegel, R., Naishadham, D., and Jemal, A. (2013). Cancer statistics, 2013. CA Cancer J. Clin. *63*, 11–30.
2. Gatta, G., Mallone, S., van der Zwan, J.M., Trama, A., Siesling, S., and Capocaccia, R. (2013). Cancer prevalence estimates in Europe at the beginning of 2000. Ann. Oncol. *24*, 1660–1666.
3. Quaglia, A., Lillini, R., Crocetti, E., Buzzoni, C., and Vercelli, M. (2013). Incidence and mortality trends for four major cancers in the elderly and middle-aged adults: an international comparison. Surg. Oncol. *22*, e31–e38.
4. Malvezzi, M., Bertuccio, P., Levi, F., La Vecchia, C., and Negri, E. (2013). European cancer mortality predictions for the year 2013. Ann. Oncol. *24*, 792–800.
5. Whang, Y.E., Armstrong, A.J., Rathmell, W.K., Godley, P.A., Kim, W.Y., Pruthi, R.S., Wallen, E.M., Crane, J.M., Moore, D.T., Grigson, G., et al. (2013). A phase II study of lapatinib, a dual EGFR and HER-2 tyrosine kinase inhibitor, in patients with castration-resistant prostate cancer. Urol. Oncol. *31*, 82–86.
6. Scher, H.I., Fizazi, K., Saad, F., Taplin, M.E., Sternberg, C.N., Miller, K., de Wit, R., Mulders, P., Chi, K.N., Shore, N.D., et al. (2012). Increased survival with enzalutamide in prostate cancer after chemotherapy. N. Engl. J. Med. *367*, 1187–1197.
7. Kalluri, R., and Weinberg, R.A. (2009). The basics of epithelial-mesenchymal transition. J. Clin. Invest. *119*, 1420–1428.
8. Dongre, A., and Weinberg, R.A. (2019). New insights into the mechanisms of epithelial-mesenchymal transition and implications for cancer. Nat. Rev. Mol. Cell Biol. *20*, 69–84.
9. Francou, A., and Anderson, K.V. (2020). The epithelial-to-mesenchymal transition in development and cancer. Annu. Rev. Cancer Biol. *4 4*, 197–220.
10. Brabletz, T., Kalluri, R., Nieto, M.A., and Weinberg, R.A. (2018). EMT in cancer. Nat. Rev. Cancer *18*, 128–134.
11. Montanari, M., Rossetti, S., Cavaliere, C., D'Aniello, C., Malzone, M.G., Vanacore, D., Di Franco, R., La Mantia, E., Iovane, G., Piscitelli, R., et al. (2017). Epithelial-mesenchymal transition in prostate cancer: an overview. Oncotarget *8*, 35376–35389.

12. Munkley, J., Li, L., Krishnan, S.R.G., Hysenaj, G., Scott, E., Dalgliesh, C., Oo, H.Z., Maia, T.M., Cheung, K., Ehrmann, I., et al. (2019). Androgen-regulated transcription of ESRP2 drives alternative splicing patterns in prostate cancer. Elife *8*, e47678.

13. De Craene, B., and Berx, G. (2013). Regulatory networks defining EMT during cancer initiation and progression. Nat. Rev. Cancer *13*, 97–110.

14. Warzecha, C.C., and Carstens, R.P. (2012). Complex changes in alternative pre-mRNA splicing play a central role in the epithelial-to-mesenchymal transition (EMT). Semin. Cancer Biol. *22*, 417–427.

15. Shah, K., Gagliano, T., Garland, L., O'Hanlon, T., Bortolotti, D., Gentili, V., Rizzo, R., Giamas, G., and Dean, M. (2020). Androgen receptor signaling regulates the transcriptome of prostate cancer cells by modulating global alternative splicing. Oncogene *39*, 6172–6189.

16. Grose, R., and Dickson, C. (2005). Fibroblast growth factor signaling in tumorigenesis. Cytokine Growth Factor. Rev. *16*, 179–186.

17. Yan, G., Fukabori, Y., McBride, G., Nikolaropolous, S., and McKeehan, W.L. (1993). Exon switching and activation of stromal and embryonic fibroblast growth factor (FGF)-FGF receptor genes in prostate epithelial cells accompany stromal independence and malignancy. Mol. Cell Biol *13*, 4513–4522.

18. Carstens, R.P., Eaton, J.V., Krigman, H.R., Walther, P.J., and Garcia-Blanco, M.A. (1997). Alternative splicing of fibroblast growth factor receptor 2 (FGF-R2) in human prostate cancer. Oncogene *15*, 3059–3065.

19. Bernard-Pierrot, I., Ricol, D., Cassidy, A., Graham, A., Elvin, P., Caillault, A., Lair, S., Broet, P., Thiery, J.P., and Radvanyi, F. (2004). Inhibition of human bladder tumour cell growth by fibroblast growth factor receptor 2b is independent of its kinase activity. Involvement of the carboxy-terminal region of the receptor. Oncogene *23*, 9201–9211.

20. Ishiwata, T. (2018). Role of fibroblast growth factor receptor-2 splicing in normal and cancer cells. Front. Biosci. *23*, 626–639.

21. Matsuda, Y., Hagio, M., Seya, T., and Ishiwata, T. (2012). Fibroblast growth factor receptor 2 IIIc as a therapeutic target for colorectal cancer cells. Mol. Cancer Ther. *11*, 2010–2020.

22. Ishiwata, T., Matsuda, Y., Yamamoto, T., Uchida, E., Korc, M., and Naito, Z. (2012). Enhanced expression of fibroblast growth factor receptor 2 IIIc promotes human pancreatic cancer cell proliferation. Am. J. Pathol. *180*, 1928–1941.

23. Zhao, Q., Caballero, O.L., Davis, I.D., Jonasch, E., Tamboli, P., Yung, W.K., Weinstein, J.N., Kenna Shaw for, T.r.n., Strausberg, R.L., and Yao, J. (2013). Tumor-specific isoform switch of the fibroblast growth factor receptor 2 underlies the mesenchymal and malignant phenotypes of clear cell renal cell carcinomas. Clin. Cancer Res. *19*, 2460–2472.

24. Oltean, S., Febbo, P.G., and Garcia-Blanco, M.A. (2008). Dunning rat prostate adenocarcinomas and alternative splicing reporters: powerful tools to study epithelial plasticity in prostate tumors in vivo. Clin. Exp. Metastasis *25*, 611–619.

25. Phan, N.N., Wang, C.Y., Chen, C.F., Sun, Z.D., Lai, M.D., and Lin, Y.C. (2017). Voltage-gated calcium channels: novel targets for cancer therapy. Oncol. Lett. *14*, 2059–2074.

26. Xiao, X., Li, B.X., Mitton, B., Ikeda, A., and Sakamoto, K.M. (2010). Targeting CREB for cancer therapy: friend or foe. Curr. Cancer Drug Targets *10*, 384–391.

27. Deliot, N., and Constantin, B. (2015). Plasma membrane calcium channels in cancer: alterations and consequences for cell proliferation and migration. Biochim. Biophys. Acta-Biomembranes *1848*, 2512–2522.

28. Roehrl, M.H.A., Kang, S.H., Aramburu, J., Wagner, G., Rao, A., and Hogan, P.G. (2004). Selective inhibition of calcineurin-NFAT signaling by blocking protein-protein interaction with small organic molecules. Proc. Natl. Acad. Sci. United States America *101*, 7554–7559.

29. Qin, J.J., Nag, S., Wang, W., Zhou, J.W., Zhang, W.D., Wang, H., and Zhang, R.W. (2014). NFAT as cancer target: mission possible? Biochim. Biophys. Acta *1846*, 297–311.

30. Xie, F.C., Li, B.X.B., Kassenbrock, A., Xue, C.H., Wang, X.Y., Qian, D.Z., Sears, R.C., and Xiao, X.S. (2015). Identification of a potent inhibitor of CREB-mediated gene transcription with efficacious in vivo anticancer activity. J. Med. Chem. *58*, 5075–5087.

31. Huang, L., Keyser, B.M., Tagmose, T.M., Hansen, J.B., Taylor, J.T., Zhuang, H., Zhang, M., Ragsdale, D.S., and Li, M. (2004). NNC 55-0396 [(1S,2S)-2-(2-(N-[(3-benzimidazol-2-yl)propyl]-N-methylamino)ethyl)-6-fluoro-1,2, 3,4-tetrahydro-1-isopropyl-2-naphtyl cyclopropanecarboxylate dihydrochloride]: a new selective inhibitor of T-type calcium channels. J. Pharmacol. Exp. Ther. *309*, 193–199.

32. Li, M., Hansen, J.B., Huang, L., Keyser, B.M., and Taylor, J.T. (2005). Towards selective antagonists of T-type calcium channels: design, characterization and potential applications of NNC 55-0396. Cardiovasc. Drug Rev. *23*, 173–196.

33. Kwok, T.C., Ricker, N., Fraser, R., Chan, A.W., Burns, A., Stanley, E.F., McCourt, P., Cutler, S.R., and Roy, P.J. (2006). A small-molecule screen in C. elegans yields a new calcium channel antagonist. Nature *441*, 91–95.

34. Kim, P.S., and Fishman, M.A. (2020). Low-Dose naltrexone for chronic pain: update and systemic review. Curr. Pain Headache Rep. *24*, 64.

35. Buchanan, P.J., and McCloskey, K.D. (2016). CaV channels and cancer: canonical functions indicate benefits of repurposed drugs as cancer therapeutics. Eur. Biophys. J. *45*, 621–633.

36. Dziegielewska, B., Gray, L.S., and Dziegielewski, J. (2014). T-type calcium channels blockers as new tools in cancer therapies. Pflugers Arch. *466*, 801–810.

37. Tajada, S., and Villalobos, C. (2020). Calcium permeable channels in cancer hallmarks. Front. Pharmacol. *11*, 968.

38. Deliot, N., and Constantin, B. (2015). Plasma membrane calcium channels in cancer: alterations and consequences for cell proliferation and migration. Biochim. Biophys. Acta *1848*, 2512–2522.

39. Figiel, S., Bery, F., Chantome, A., Fontaine, D., Pasqualin, C., Maupoil, V., Domingo, I., Guibon, R., Bruyere, F., Potier-Cartereau, M., et al. (2019). A novel calcium-mediated EMT pathway controlled by lipids: an opportunity for prostate cancer adjuvant therapy. Cancers (Basel) *11*, 1814.

40. Zylla, D., Gourley, B.L., Vang, D., Jackson, S., Boatman, S., Lindgren, B., Kuskowski, M.A., Le, C., Gupta, K., and Gupta, P. (2013). Opioid requirement, opioid receptor expression, and clinical outcomes in patients with advanced prostate cancer. Cancer *119*, 4103–4110.

41. Silvestri, R., Pucci, P., Venalainen, E., Matheou, C., Mather, R., Chandler, S., Aceto, R., Rigas, S.H., Wang, Y., Rietdorf, K., et al. (2019). T-type calcium channels drive the proliferation of androgen-receptor negative prostate cancer cells. Prostate *79*, 1580–1586.

42. Park, S.H., Park, S.J., Kim, J.O., Shin, J.H., Kim, E.S., Jo, Y.K., Kim, J.S., Park, S.J., Jin, D.H., Hwang, J.J., et al. (2013). Down-regulation of survivin by nemadipine-A sensitizes cancer cells to TRAIL-induced apoptosis. Biomol. Ther. (Seoul) *21*, 29–34.

43. Zagon, I.S., and McLaughlin, P.J. (1983). Naltrexone modulates tumor response in mice with neuroblastoma. Science *221*, 671–673.

44. Liu, N., Ma, M., Qu, N., Wang, R., Chen, H., Hu, F., Gao, S., and Shan, F. (2020). Low-dose naltrexone inhibits the epithelial-mesenchymal transition of cervical cancer cells in vitro and effects indirectly on tumor-associated macrophages in vivo. Int. Immunopharmacol. *86*, 106718.

45. Oltean, S., and Bates, D.O. (2014). Hallmarks of alternative splicing in cancer. Oncogene *33*, 5311–5318.

46. Yasumoto, H., Matsubara, A., Mutaguchi, K., Usui, T., and McKeehan, W.L. (2004). Restoration of fibroblast growth factor receptor2 suppresses growth and tumorigenicity of malignant human prostate carcinoma PC-3 cells. Prostate *61*, 236–242.

47. Teishima, J., Hayashi, T., Nagamatsu, H., Shoji, K., Shikuma, H., Yamanaka, R., Sekino, Y., Goto, K., Inoue, S., and Matsubara, A. (2019). Fibroblast growth factor family in the progression of prostate cancer. J. Clin. Med. *8*, 183.

48. Wang, B.D., Ceniccola, K., Hwang, S., Andrawis, R., Horvath, A., Freedman, J.A., Olender, J., Knapp, S., Ching, T., Garmire, L., et al. (2017). Alternative splicing promotes tumour aggressiveness and drug resistance in African American prostate cancer. Nat. Commun. *8*, 15921.

49. Jimenez-Vacas, J.M., Herrero-Aguayo, V., Montero-Hidalgo, A.J., Gomez-Gomez, E., Fuentes-Fayos, A.C., Leon-Gonzalez, A.J., Saez-Martinez, P., Alors-Perez, E., Pedraza-Arevalo, S., Gonzalez-Serrano, T., et al. (2020). Dysregulation of the splicing machinery is directly associated to aggressiveness of prostate cancer. EBioMedicine *51*, 102547.

50. Olender, J., and Lee, N.H. (2019). Role of alternative splicing in prostate cancer aggressiveness and drug resistance in african Americans. Adv. Exp. Med. Biol. *1164*, 119–139.

51. Davis, F.M., Stewart, T.A., Thompson, E.W., and Monteith, G.R. (2014). Targeting EMT in cancer: opportunities for pharmacological intervention. Trends Pharmacol. Sci. *35*, 479–488.

52. Lahat, G., Zhu, Q.S., Huang, K.L., Wang, S.Z., Bolshakov, S., Liu, J., Torres, K., Langley, R.R., Lazar, A.J., Hung, M.C., et al. (2010). Vimentin is a novel anti-cancer therapeutic target; insights from in vitro and in vivo mice xenograft studies. PLoS One *5*, e10105.

53. Tanaka, H., Kono, E., Tran, C.P., Miyazaki, H., Yamashiro, J., Shimomura, T., Fazli, L., Wada, R., Huang, J.T., Vessella, R.L., et al. (2010). Monoclonal antibody targeting of N-cadherin inhibits prostate cancer growth, metastasis and castration resistance. Nat. Med. *16*, 1414–U1496.

OMTM, Volume *25*

Supplemental information

A repositioning screen using an FGFR2 splicing reporter reveals compounds that regulate epithelial-mesenchymal transitions and inhibit growth of prostate cancer xenografts

Ling Li, Jinxia Zheng, Megan Stevens, and Sebastian Oltean

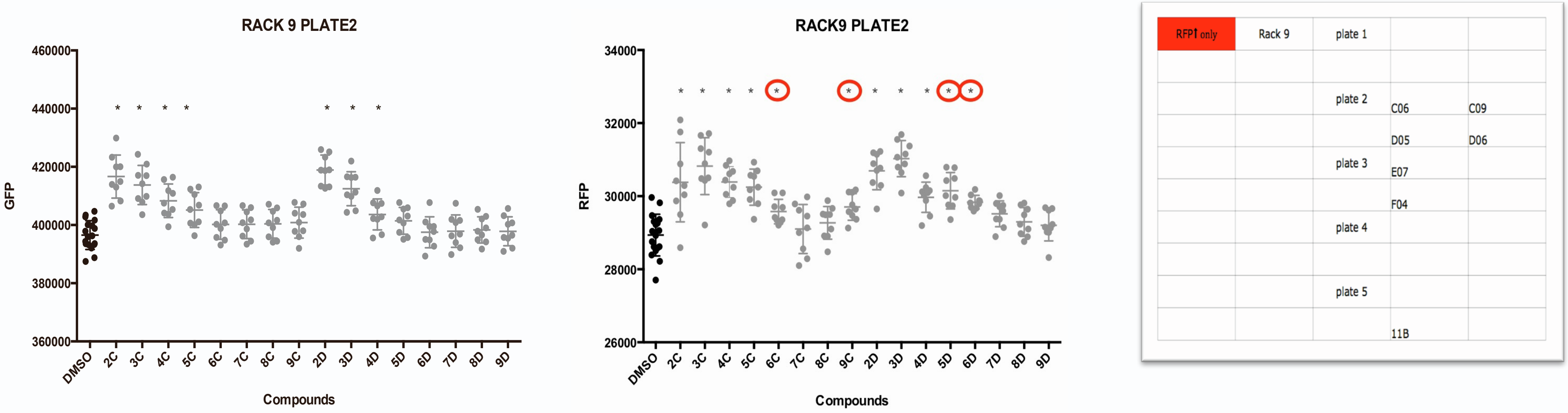

Supplementary Figure 1. Examples of readings in the primary screen, that resulted in narrowing down to 278 compounds.

Left panel represent GFP read-outs from the plate reader and data analysed by Prism.

Middle panel represent dsRED read-outs from the plate reader and data analysed by Prism, red circles marked the hits that increased dsRED without increasing GFP signal.

Right panel represent the final result, in which red means RFP increase only. X-axis represent the location in the 96-well plate. Each well represent one repeat of one chemical treatment. Each treatment had three repeats. *$p<0.05$ by One-way ANOVA using GraphPad Prism.

Primary screen result		Elimination screen	Final result
Rack 10	D07	RFP↑ only	False-positives
Rack 10	F04	RFP↑ + GFP↓	False-positives
Rack 13	B04	RFP↑ only	GFP↓ only
Rack 14	B05	RFP↑ only	False-positives
Rack 15	E10	GFP↓ only	RFP↑ only

Supplementary Figure 2. Examples of eliminating false-positive
Red means RFP increase only, green means GFP decrease only, and yellow means both of them in the primary screen. Rack10, compound D07 showed RFP increase in primary screen but also showed the same result in elimination-screen, so it was eliminated. Rack13 B04 showed both changes in the primary screen while only increased RFP in the elimination screen; therefore, the effect suggested by GFP decrease may be a real one, related to splicing and not a false-positive.

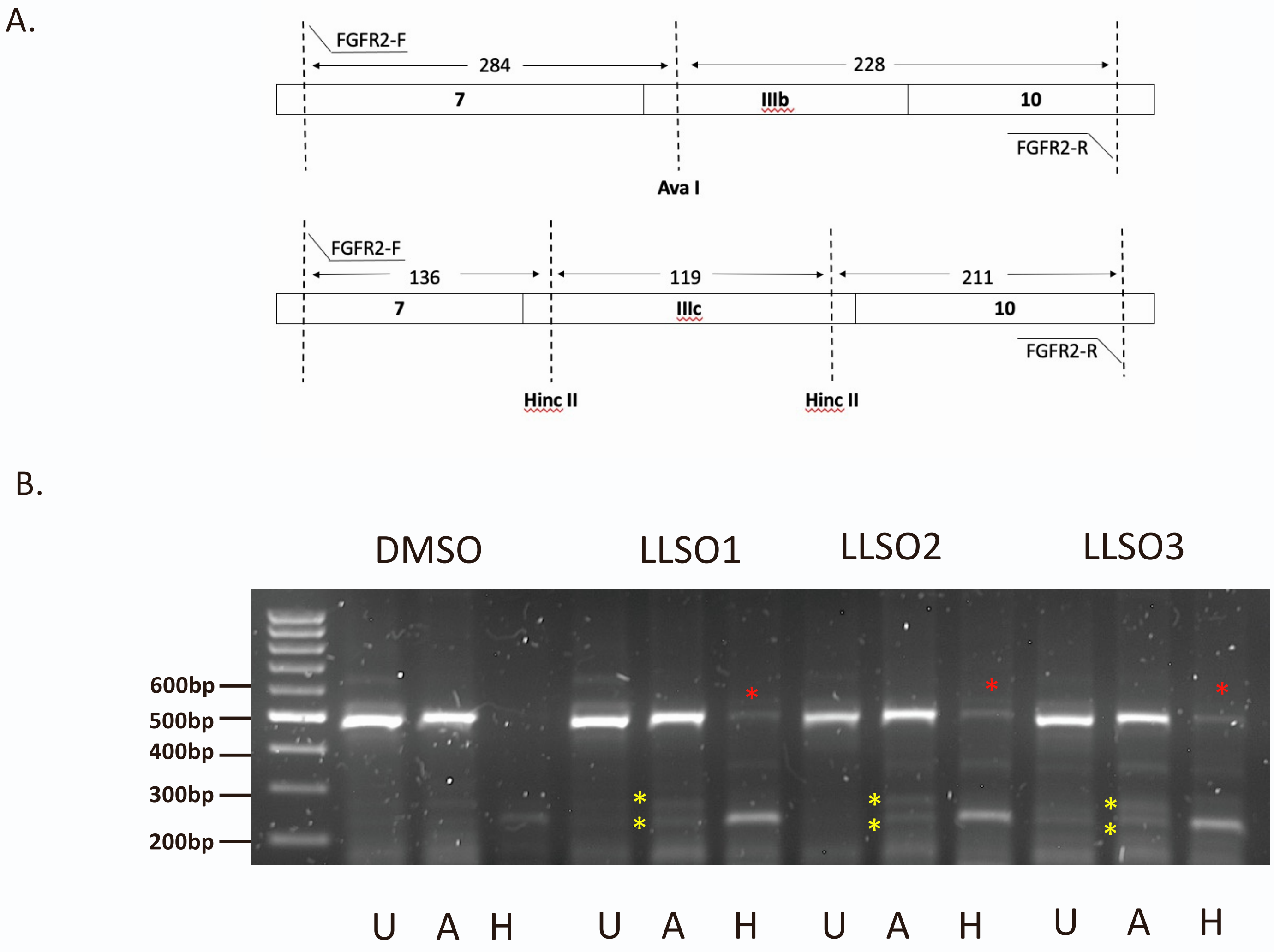

Supplementary Figure 3. The same analysis as Figure 2 (see legend) but done in PC3 prostate cancer cells instead of HEK293

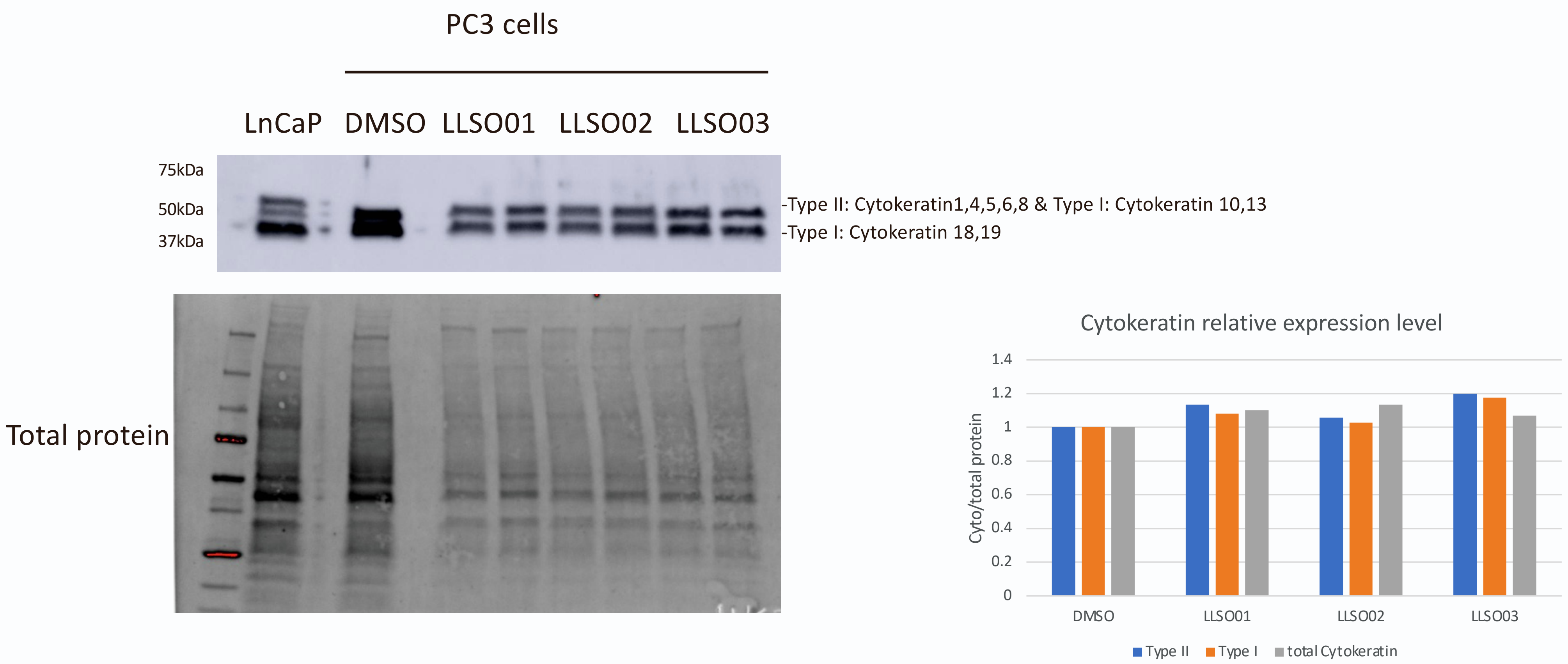

Supplementary Figure 4. Response of additional markers of EMT to LLSOs treatment - cytokeratins. Western blot analysis showing a slight increase in cytokeratins expression in PC3 cells exposed to LLSOs treatments for 48 h at 10uM (n=2 replicates, 1experiment).

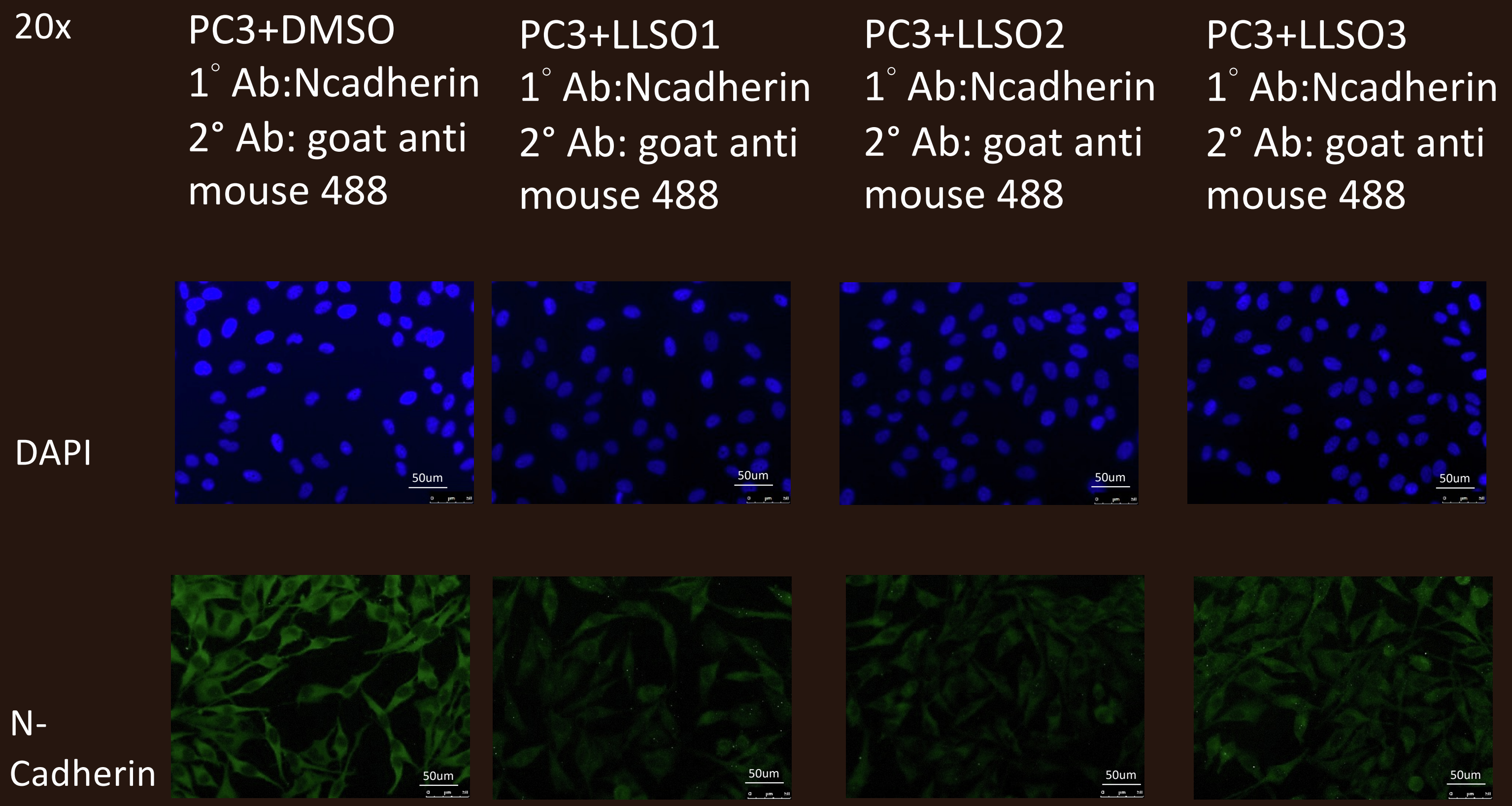

Supplementary Figure 5. Immunofluorescence analysis of PC3 cells treated with LLSO1, LLSO2 or LLSO3 at 10 uM for 48 hours shows a decrease in expression of N-cadherin. Representative examples from at least 10 microscopic fields per experiment, and experiments repeated 3 times. Pictures taken at 200 X magnification.

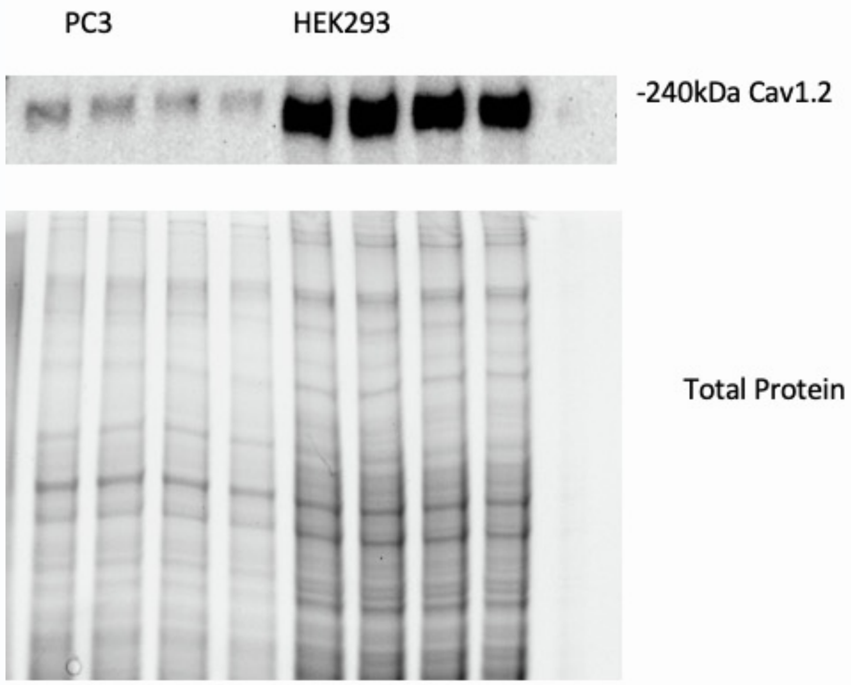

Supplementary Figure 6. L-type Ca channels are expressed in PC3 prostate cancer cells

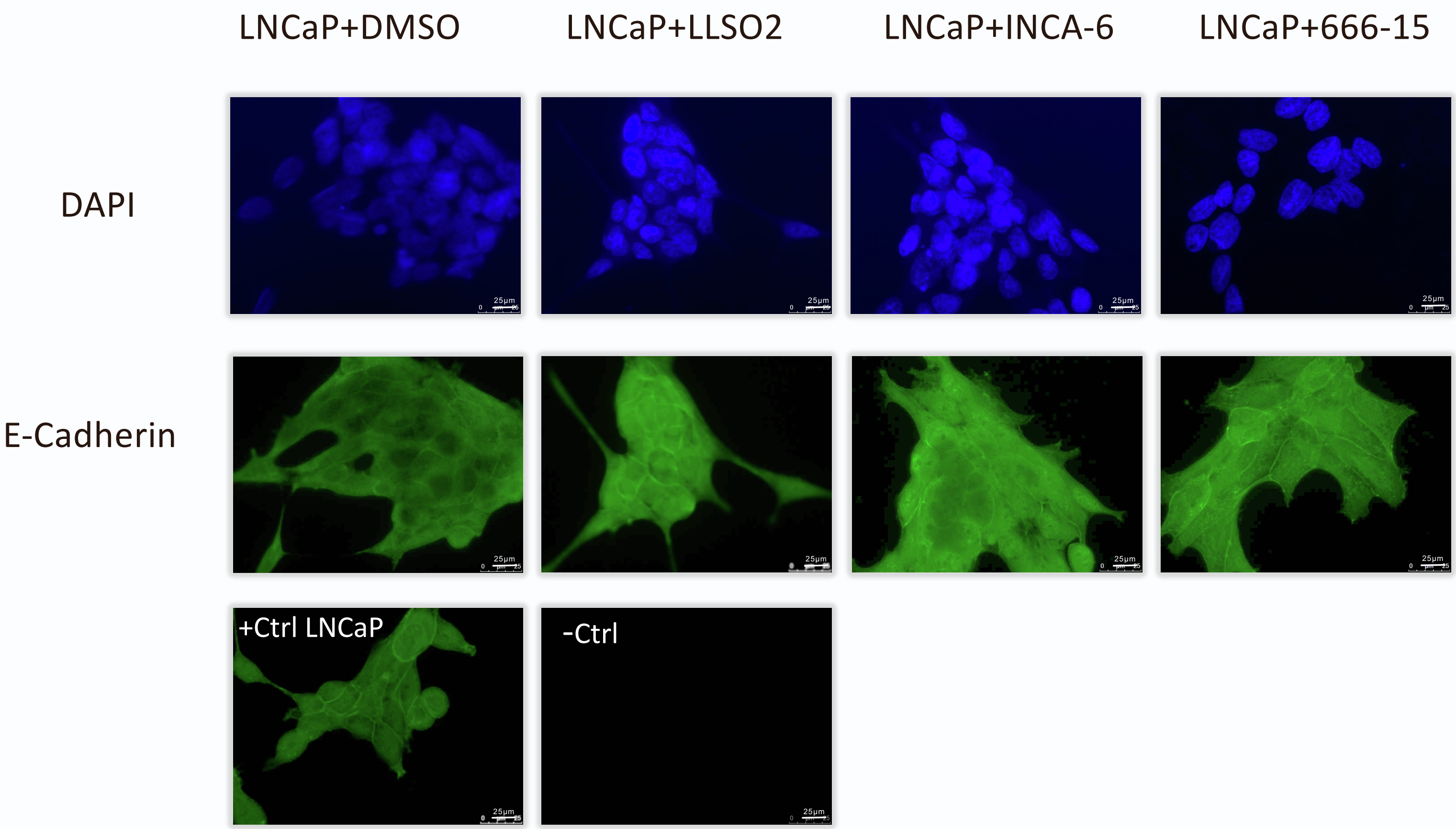

Supplementary Figure 7. LLSO02 uses NFAT and/or CREB to signal to increase E-cadherin expression in LNCaP cells
Images of LNCaP cells treated separately with 10μM of LLSO2, 10μM of INCA-6, and 10μM of 666-15
All images are taken at 20x magnification.

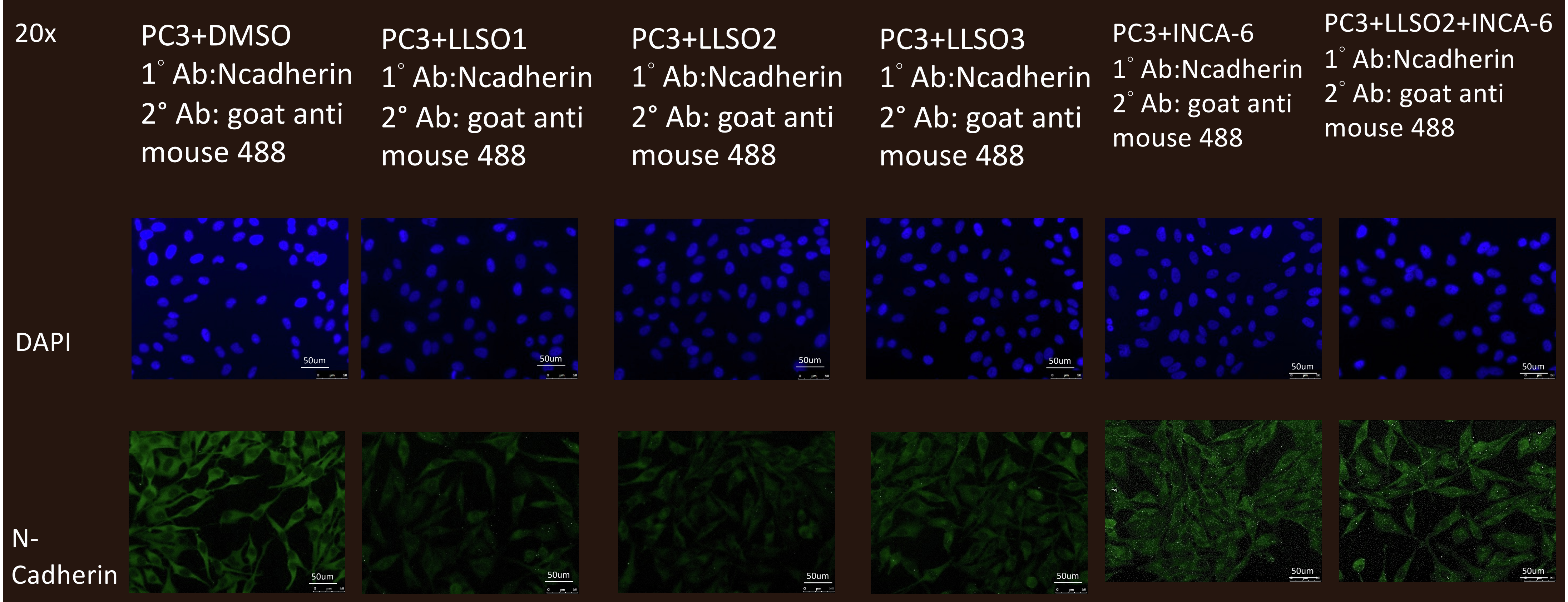

Supplementary Figure 8. Immunofluorescence analysis of PC3 cells treated with LLSO1, LLSO2 or LLSO3 at 10 uM for 48 hours shows a decrease in expression of N-cadherin (the examples shown here are the same as the ones shown in Supplementary Figure 5 above). When INCA-6 is used or INCA-6 and LLSO2, N-cadherin expression is rescued. Representative examples from at least 10 microscopic fields per experiment, and experiments repeated 3 times. Pictures taken at 200 X magnification.

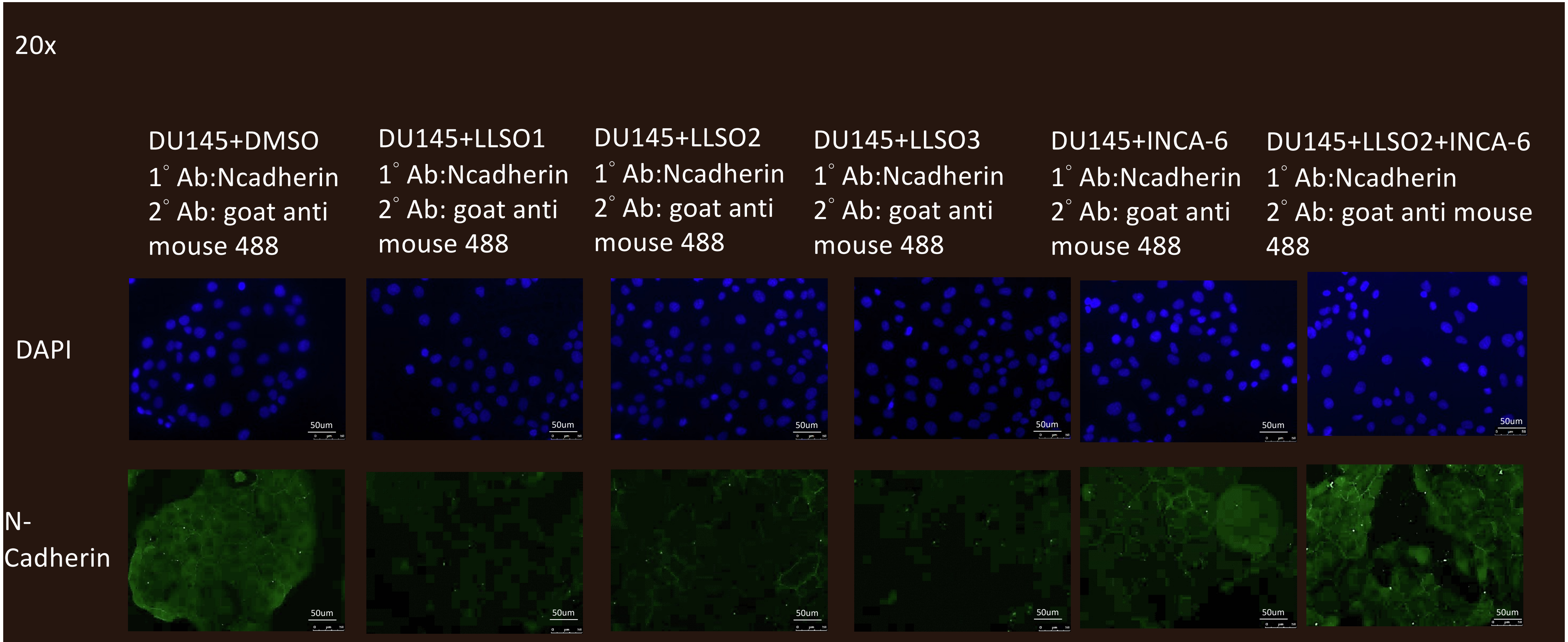

Supplementary Figure 9. Immunofluorescence analysis of DU145 cells treated with LLSO1, LLSO2 or LLSO3 at 10 uM for 48 hours shows a decrease in expression of N-cadherin. When INCA-6 is used or INCA-6 and LLSO2, N-cadherin expression is rescued. Representative examples from at least 10 microscopic fields per experiment, and experiments repeated 3 times Pictures taken at 200 X magnification.

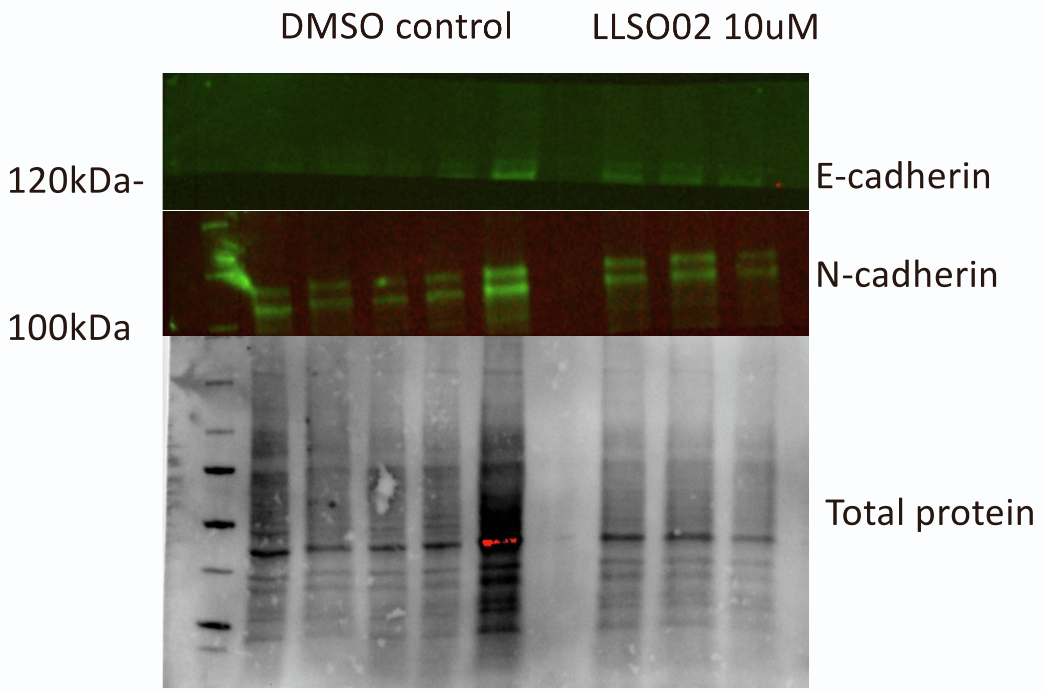

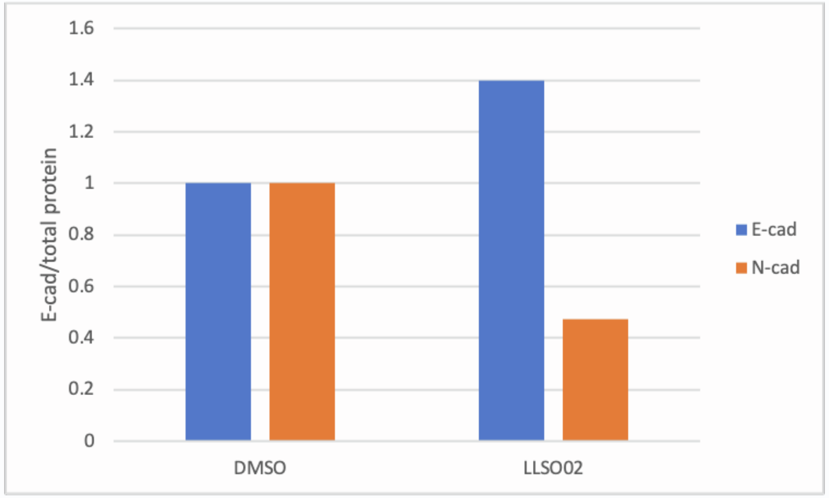

Supplementary Figure 10. LLSO02 induces MET changes in tumour xenografts. Western blot analysis of protein extracts from excised tumours show an increase in E-cadherin and a decrease in N-cadherin, consistent with inducing MET (n=3 replicates, 1 experiment).

LC50 for PC3 treated with LLSO1

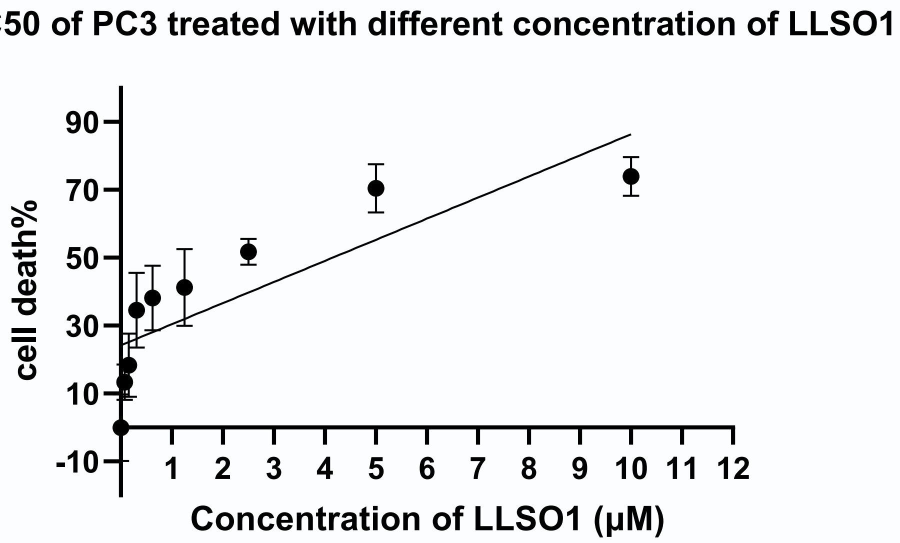

Y = 6.216*X + 24.22

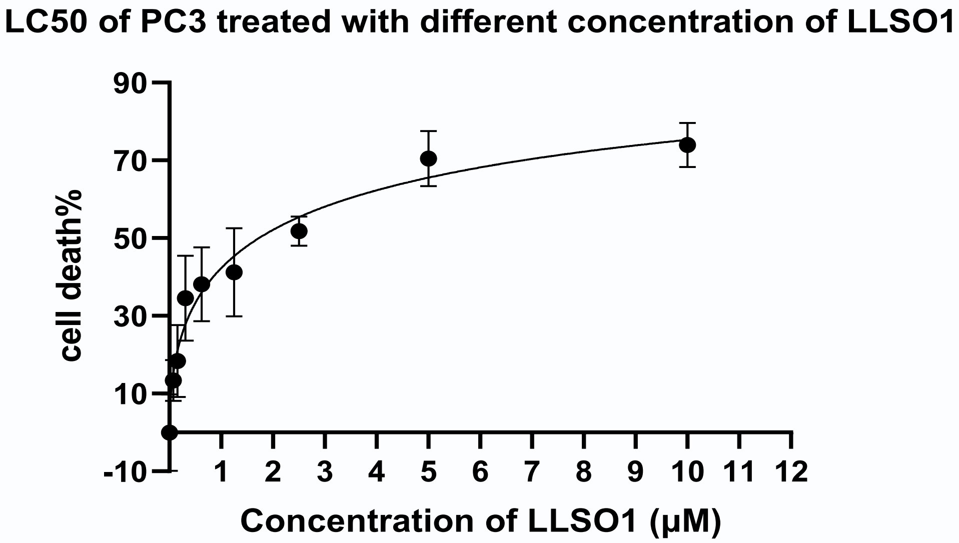

Y = 6.216*X + 24.22

Y = mx + c,   Y = 6.216*X + 24.22,   m = 6.216, c = 24.22

According to LC50 = [(50-c)/m] formula, LC50 for PC3 treated with LLSO1 is (50-24.22)/6.216 = 4.15μM

Supplementary Figure 11. The LC50 curve of PC3 treated with different concentration of LLSO1.
MTT assay was performed following 48 hours pre-treatment of DMSO and different concentration of LLSO1 in PC3 cells. Absorbance rate on MTT assay of PC3 treated with 0.078125μM, 0.15625μM, 0.3125μM, 0.625μM, 1.25μM, 2.5μM, 5μM, 10μM LLSO01, and DMSO (as control) at OD = 590nm. Six replicated within each experiment. The LC50 for PC3 treated with LLSO1 is 4.15μM

Leo Simanjuntak et al 2018 IOP Conf. Ser.: Mater. Sci. Eng. 420 012144

LC50 for PC3 treated with LLSO2

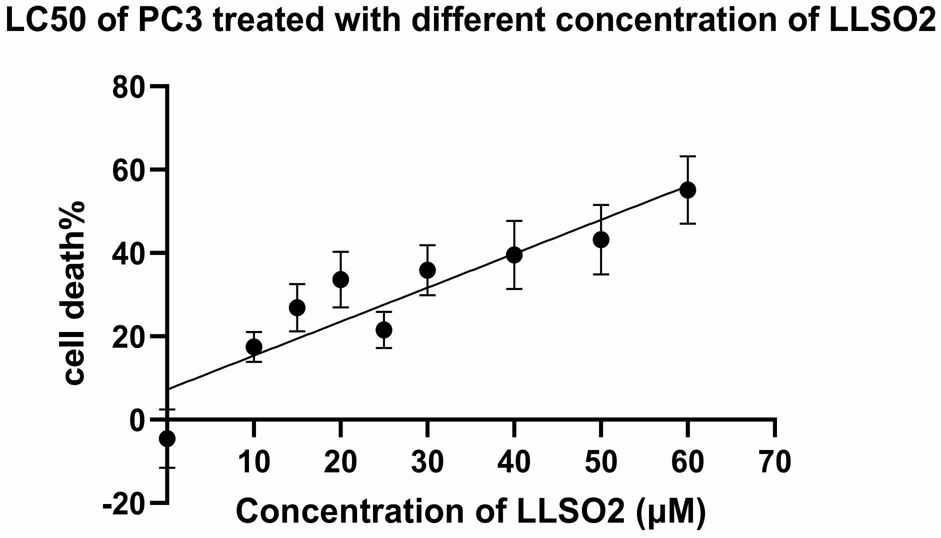

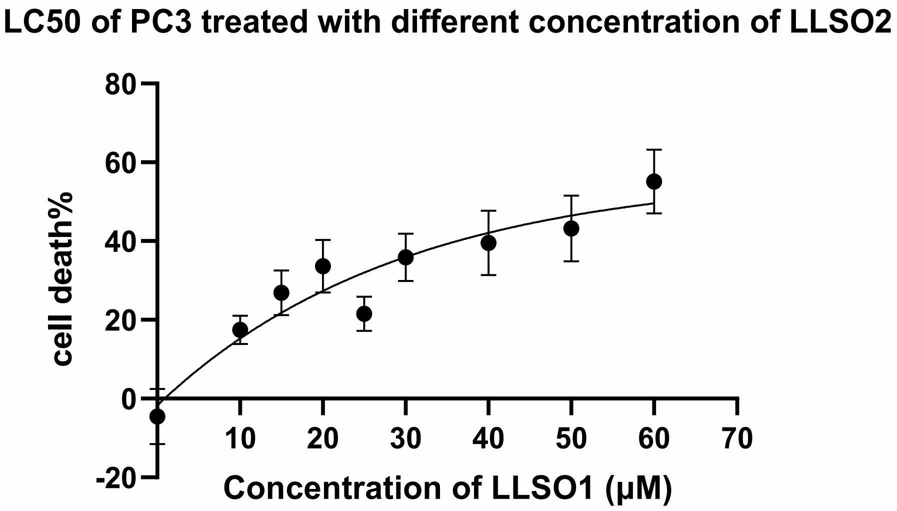

Y = 0.8152*X + 7.220

Y = 0.8152*X + 7.22

Y = mx + c,   Y = 0.8152*X + 7.22,   m = 0.8152, c = 7.22

According to LC50 = [(50-c)/m] formula, LC50 for PC3 treated with LLSO1 is (50-7.22)/0.8152 = 52.48μM

Supplementary Figure 12. The LC50 curve of PC3 treated with different concentration of LLSO2. MTT assay was performed following 48 hours pre-treatment of DMSO and different concentration of LLSO2 in PC3 cells. Absorbance rate on MTT assay of PC3 treated with 10μM, 15μM, 20μM, 25μM, 30μM, 40μM, 50μM, 60μM LLSO02, and DMSO (as control) at OD = 590nm. Six replicated within each experiment. The LC50 for PC3 treated with LLSO2 is 52.48μM

LC50 for PC3 treated with LLSO3

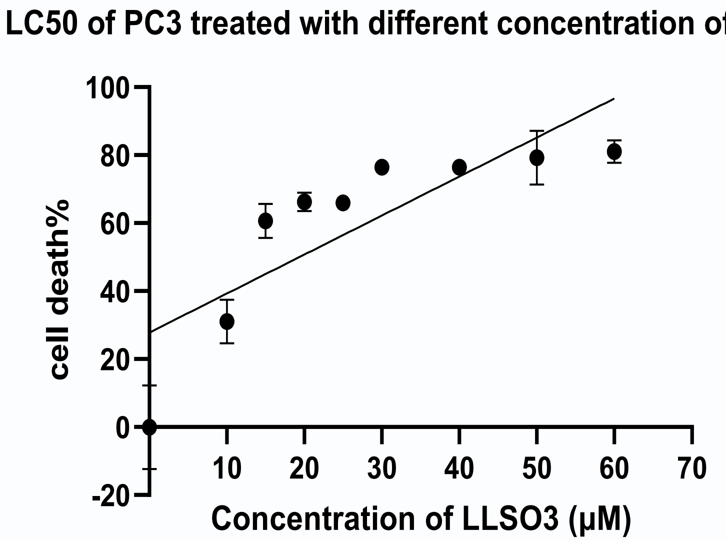

Y = 1.148*X + 27.79

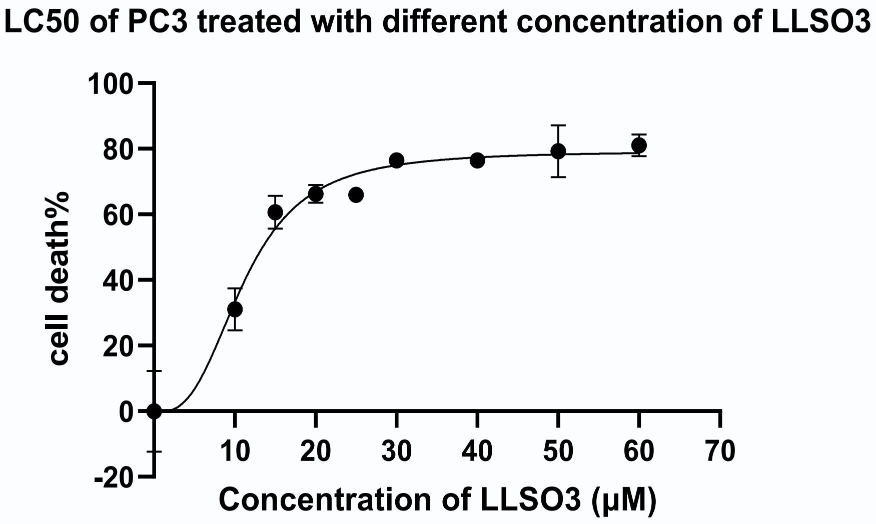

Y = 1.148*X + 27.79

Y = mx + c,   Y = 1.148*X + 27.79,   m = 1.148, c = 27.79

According to LC50 = [(50-c)/m] formula, LC50 for PC3 treated with LLSO1 is (50-27.79)/1.148 = 19.35μM

Supplementary Figure 13. The LC50 curve of PC3 treated with different concentration of LLSO3.
MTT assay was performed following 48 hours pre-treatment of DMSO and different concentration of LLSO3 in PC3 cells. Absorbance rate on MTT assay of PC3 treated with 10μM, 15μM, 20μM, 25μM, 30μM, 40μM, 50μM, 60μM LLSO03, and DMSO (as control) at OD = 590nm. Six replicated within each experiment. The LC50 for PC3 treated with LLSO3 is 19.35μM
